# Microwave-Assisted Sequential One-Pot Synthesis of 8-Substituted Pyrazolo[1,5-*a*][1,3,5]triazines

**DOI:** 10.3390/molecules26123540

**Published:** 2021-06-10

**Authors:** Jonathan Elie, Corinne Fruit, Thierry Besson

**Affiliations:** 1 Perha Pharmaceuticals, Perharidy Peninsula, 29680 Roscoff, France; elie@perha-pharma.com (J.E.); corinne.fruit@univ-rouen.fr (C.F.); 2 Normandie University, UNIROUEN, INSA Rouen, CNRS, COBRA UMR 6014, 76000 Rouen, France

**Keywords:** microwave-assisted chemistry, one-pot synthesis, pyrazolo[1,5-*a*][1,3,5]triazines, 5-aza-9-deazapurines

## Abstract

This paper reports a convenient sequential one-pot approach for the synthesis of an array of 14 pyrazolo[1,5-*a*][1,3,5]triazines, substituted in C8 by halogen (Br), various functions (CN and CO_2_Et) and alkyl or (het)aryl groups. This study confirms the interest of combining the efficient heating obtained under dielectric microwave heating and the achievement of sequential one-pot reactions, avoiding the tedious work-up and purification of intermediate compounds, achieving sustainable synthesis processes. Considering usual conventional methods, this microwave protocol is featured by advantages in terms of yields, reaction times, and convenient gram scale synthesis.

## 1. Introduction

**Figure 1:**
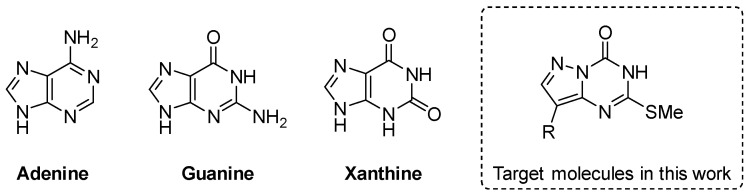
Structures of biogenic purines (adenosine, guanine, xanthine) and C8-functionalized 2-(methylsulfanyl)pyrazolo[1,5-*a*]-1,3,5-triazin-4(3*H*)-ones targeted in this study.

The pyrazolo[1,5-*a*][1,3,5]triazine is a heterocyclic system which has generated considerable interest in the communities of academic and industrial medicinal chemists [1,2]. This nitrogen-rich heterocyclic core was intensively developed as a bioisosteric substitute of biogenic purines (e.g., adenine, guanine, and xanthine in Figure 1) for the conception of potentially bioactive compounds in a wide range of biological applications [3,4,5,6,7,8,9,10,11,12,13,14,15,16,17,18,19,20,21]. In this context, numerous synthetic routes of various substituted pyrazolo[1,5-*a*][1,3,5]triazines have been yet reported [22,23,24,25,26,27,28,29,30,31,32,33,34,35,36,37]. Extending our interest in associating microwave-assisted chemistry with one-pot sequential approaches [38,39,40], we envisioned developing convenient and sustainable access to C8-functionalized 2-(methylsulfanyl)pyrazolo[1,5-*a*]-1,3,5-triazin-4(3*H*)-ones that are privileged precursors to complex molecules at therapeutic aims (Figure 1).

This paper reports a convenient one-pot process for the synthesis of these 5-aza-9-deazapurines substituted in C8 by various functions (CN and CO_2_Et) and alkyl or (het)aryl groups.

## 2. Results and Discussion

**Scheme 1:**
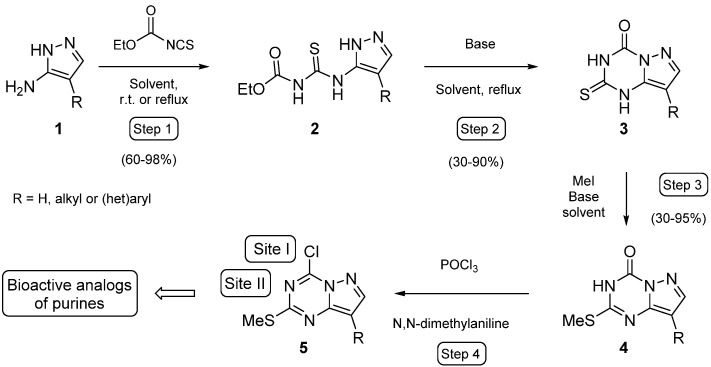
Usual synthetic routes of the target heterocyclic systems (**4**). The range of yields described for steps 1, 2 and 3, can vary according to the various protocols.

Based on literature data, three steps are required for the synthesis of the target heterocyclic systems. The first step started from 5-aminopyrazole or its 4-substituted analogs (**1**) which were reacted with ethoxycarbonyl isothiocyanate in various solvents (e.g., ethyl acetate, [10], ethyl acetate/benzene [13,21] or dimethylformamide DMF [14]) to give the intermediate *N*-carbetoxythioureas (**2**). The latest were heated in basic conditions (e.g., ammonium hydroxide in methanol [10], aqueous sodium hydroxide [13,14,15,21,22,25,29], sodium methoxide [5] and sodium ethoxide in ethanol [7,14,15]) and then cyclized, giving the 2-thioxo-1*H*-pyrazolo[1,5-*a*][1,3,5]triazin-4-ones (**3**). The following S-methylation with methyl iodide (MeI) in the presence of sodium hydroxide (NaOH) gave the corresponding 2-(methylsulfany)lpyrazolo[1,5-*a*][1,3,5]triazin-4(3*H*)-one derivatives (**4**) [10,13,14,15,21,22,23]. In various works, chlorination of compounds **4** with phosphorus oxychloride (POCl_3_), in the presence of *N*,*N*-dimethylaniline [13,14,15] gave 4-chloro-2-methylthio-[1,5-*a*][1,3,5]triazines (**5**), highly versatile molecular bricks featuring two main substitution sites (labeled site I and site II in Scheme 1) allowing a wide range of ring extensions in the case of scaffold hopping strategies.

**Scheme 2:**
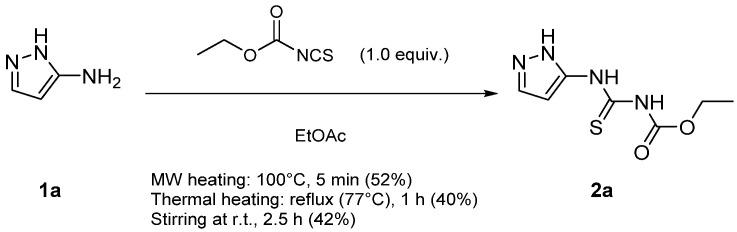
Comparison of MW-assisted vs. conventional thermal heating for the synthesis of *N*-carbetoxythiourea (**2a**).

In an initial attempt to perform a microwave-assisted process for the first step, a mixture of 5-aminopyrazole (**1a**, R = H) and ethoxycarbonylisothiocyanate (1 equiv.) in ethyl acetate (AcOEt) was heated in sealed vial under microwaves at 100 °C for 5 min and afforded the corresponding *N*-carbetoxythiourea (**2a**) in a 52% yield (Scheme 2).

Applying conventional heating methods previously described at atmospheric pressure (reflux 77 °C, 1 h in an oil bath), compound **2a** was isolated in only 40% yield, while 84% was reported in the literature [17]. The reaction was repeated and the yield remained unchanged. Whatever methods applied it was found that the work-up and isolation of the product was trickier than described, and degradation products (not identified) were found at the end of the process while crude ^1^H-NMR analysis indicated a complete conversion of the starting pyrazole.

**Scheme 3:**
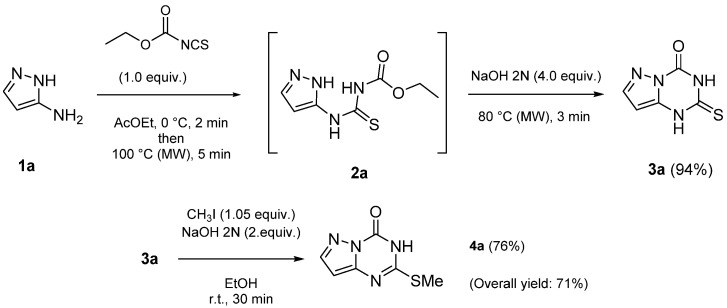
MW-assisted sequential one-pot synthesis of **3a** and its *S*-methylation into **4a**.

To overcome such low yields and tedious work-up, the synthesis of 2-thioxo-1*H*-pyrazolo[1,5-*a*][1,3,5]triazin-4-one (**3a**) was envisioned from **1a** via a one-pot approach. The development of the experimental conditions confirmed the necessity to add slowly the isothiocyanate derivative at 0 °C (0.2 mL/min) before irradiating the sealed microwave vial [21,22]. After 5 min of heating at 100 °C, EtOAc was evaporated and aqueous NaOH (2N) added. The resulting suspension was then stirred for 3 min at 80 °C under microwave irradiation. After work-up, the expected product **3a** was isolated by filtration, in an excellent yield (94%) (Scheme 3). Methylation of the sulfur atom of **3a** was realized with iodomethane (MeI) in 30 min at room temperature (r.t.) in basic conditions (NaOH 2N), with ethanol (EtOH) as solvent, affording 2-(methylsulfanyl)[1,3,5]triazin-4(3*H*)-one derivative (**4a**) in a 76% yield, and in an overall yield of 71% from 5-aminopyrazole (**1a**) (Scheme 3).

The preparation of **4a** and its derivatives is a crucial step in the synthesis of multi-functionalized compounds that are key precursors for further bioactive compounds. After considering the experimental conditions described above, the whole process was carried out in the same vessel, avoiding intermediate purification of **2a** and **3a** and introducing the reagents sequentially. In this novel procedure, tetrahydrofuran (THF) replaced ethyl acetate. This solvent is inert to the reaction conditions and improve the solubilization of reaction intermediates.

**Scheme 4:**
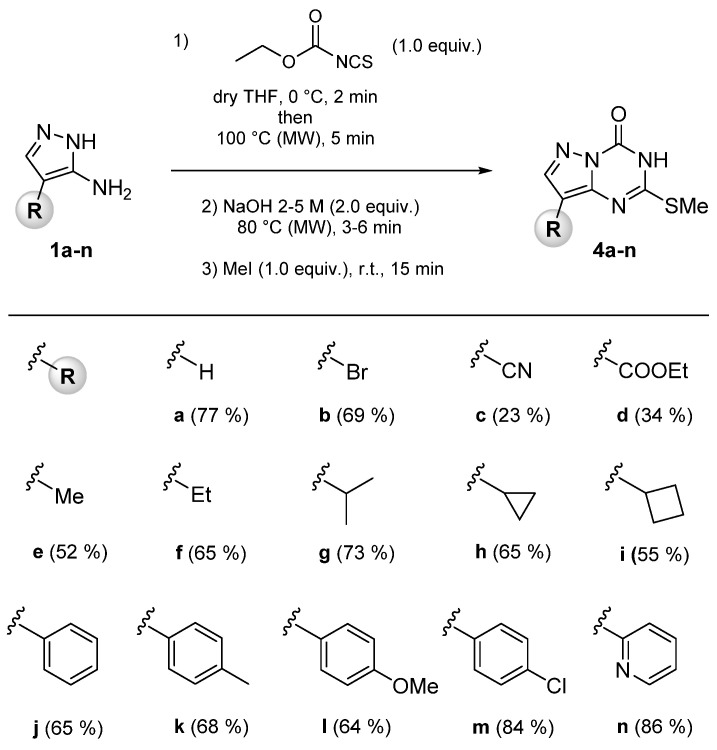
Scope of the MW-assisted sequential process giving 2-(methylsulfanyl)pyrazolo[1,5-*a*][1,3,5]triazin-4(3*H*)-ones (**4a**–**n)** from 5-aminopyrazoles (**1a**–**n**).

Specifically, in a microwave vial, ethoxycarbonyl isothiocyanate (1.0 equiv.) was added dropwise to a solution of 5-aminopyrazole (**1a**) (1.0 equiv.) in dry THF (3 mL) at 0 °C. After complete addition, the mixture was stirred 2 min at room temperature and the vessel was sealed, deposited in a microwave reactor and heated at 100 °C for 5 min. After cooling until 50 °C, NaOH 2N (2.0 equiv.) was added and the vial was sealed again and irradiated at 80 °C for 3 min. After cooling, MeI (1.0 equiv.) was added dropwise and the mixture was stirred for 15 min at room temperature. No flash chromatography on silica gel was required for the purification step and the precipitated product **4a** was isolated after acidification, filtration, washing and drying steps, as a pure white solid. With a yield of 77% (Scheme 4), this 2-(methylsulfanyl)triazine derivative was obtained in a slightly higher overall yield than reported above (71%). The scale of the reaction has been successfully enhanced, since starting from 1.0 g or 2.0 g of 5-aminopyrazole (**1a**) yielded 73% and 72% of the attempted product **4a**, respectively.

With optimized conditions in hand, the reaction conditions were applied to a range of 5-aminopyrazoles (**1b**–**n**) substituted at C8 by a halogen atom (Br) (**1b**), functional groups (carbonitrile **1c**, ethyl ester **1d**), or various alkyl (**1e**–**i**) and (het)aryl groups (**1j**–**n**). The corresponding 2-(methylsulfanyl)pyrazolo[1,5-*a*][1,3,5]triazin-4(3*H*)-ones (**4b**–**n**) were obtained as depicted in Scheme 4. Except for the two electro-withdrawing carbonitrile and ethyl carboxylate substituents for which low yields were observed, the effect of the alkyl and aryl substituents cannot be really evaluated since an average value of around 70% can be observed. It may be noted that when the same sequential one-pot procedures (sealed vessel, quantities of reactants, solvent, base and reaction times) were applied to the synthesis of compounds **4a** and **4f**, with a traditional oil bath heating system, the overall yields observed were in the 45–50% range.

**Scheme 5:**
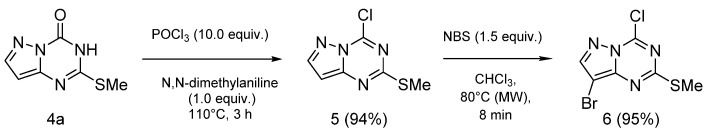
Synthesis of pyrazolo[1,5-*a*][1,3,5]triazine **6** from **4a**. In the first step, the reaction mixture was heated in conventional heating conditions (oil bath).

To complete our study, the synthesis of the polyfunctionalized 8-bromo-4-chloro-2-(methylsulfanyl)pyrazolo[1,5-*a*][1,3,5]triazine (**6**) was investigated. Considering that previous attempts of microwave-assisted chlorination of analogous pyrimidin-4-ones with POCl_3_ were revealed difficult and sometimes quite hazardous [41], C4-chlorination of compound **4a** was performed via conventional operating conditions described in various papers and patents [10,13,14,15,21,22,23]. The reaction was successfully carried out at 110 °C for 3 h in a sealed vessel using a large excess (10 equiv.) of freshly opened commercial POCl_3_, in the presence of *N*,*N*-dimethylaniline (1.0 equiv.). Intermediate 4-chloro-2-(methylsulfanyl)pyrazolo[1,5-*a*][1,3,5]triazine (**5**) was isolated in 94% yield (Scheme 5). However, the purification phase appeared to be very critical and it was noted that compound **5** may be unstable over time: it is strongly sensitive to moisture and its hydrolysis lead to its oxygenated precursor. Considering these results, synthesis of brominated product **6** was carried out from freshly isolated compound **5**, with an excess (1.5 equiv.) of *N*-bromosuccinimide (NBS). This microwave-assisted process provided **6** with an excellent yield of 95%. A scale-up was successfully achieved in the same yield, starting from 1 g of **4a** for the first step and 2 g of **5** for the second one (Scheme 5).

## 3. Materials and Methods

Compound **3**, and some derivatives of the **4** series (**4a**–**g**) were randomly described in academic works and patents cited in this paper. To complete data sometimes uneasy to found in the literature, all compounds **4a**–**n** were fully characterized. Compounds **5** and **6** were briefly described in Refs1–23]. ^1^H NMR and ^13^C NMR spectra of these compounds are available in Supplementary Materials (Section S1–S20). General information and procedure for their synthesis are described below.

### 3.1. General Information

All reagents (**1a**–**n**) were purchased from commercial suppliers (Alfa Aesar, Thermo Fisher Scientific Heysham, Lancashire, UK) or Manchester Organics Ltd. (The Heath Business & Technical Park, Runcorn WA7 4QX, UK) or Enamine Ltd. (LV-1035 Riga, Latvia) or Merck KGaA (Darmstadt, Germany), and were used without further purification. All reactions were monitored by thin-layer chromatography with aluminum plates (0.25 mm) precoated with silica gel 60 F254 (Merck KGaA Darmstadt, Germany or Fluorochem, Hadfield, Derbyshire, UK). Visualization was performed with UV light at a wavelength of 254 nm. Melting points of solid compounds were measured with a SMP3 Melting Point instrument (STUART, Bibby Scientific Ltd., Roissy, France) with a precision of 1.5 °C. IR spectra were recorded with a Spectrum 100 Series FTIR spectrometer (PerkinElmer, Villebon S/Yvette, France). Liquids and solids were investigated with a single-reflection attenuated total reflectance (ATR) accessory; the absorption bands are given in cm^−1^. NMR spectra (^1^H and ^13^C) were acquired at 295 K using an AVANCE 300 MHz or AVANCE III 400 MHz spectrometer (Bruker, Wissembourg, France) at 300 or 400 MHz for ^1^H and 75.4 or 101 MHz for ^13^C. Coupling constant J was in Hz and chemical shifts were given in ppm. Mass (ESI, EI and field desorption (FD) were recorded with an LCP 1er XR spectrometer (WATERS, Guyancourt, France)). Mass spectrometry was performed by the Mass Spectrometry Laboratory of the University of Rouen. The mass spectra [ESI, EI, and field desorption (FD)] were recorded with an LCP 1er XR spectrometer (WATERS, Guyancourt, France). Microwaves-assisted reactions were carried out in sealed tubes with a Monowave 400 instrument and temperatures were measured by IR-sensor (Anton Paar France S.A.S., les Ulis, France). Time indicated in the various protocols is the time measured when the mixtures were at the programmed temperature.

Regarding the keto-enolic tautomeric forms of these molecules (pyrazolo[1,5-*a*]-1,3,5-triazin-4(3*H*)-ones or pyrazolo[1,5-*a*][1,3,5]triazin-4-ols), complete DEPT135, and two-dimensional NMR experiments (HMBC, HSQC and NOESY) were performed with compounds **4a** and **4g** (see Supplementary Materials). Analysis of the IR investigations are rather in favor of the pyrazolo[1,5-*a*]-1,3,5-triazin-4(3*H*)-one as the privileged shape. This seems confirmed by comparing the ^1^H NMR spectra in SM Section with data given in [13] (for **4a**) and in Ref. [42] (see Supplementary Information).

### 3.2. Chemistry

#### 3.2.1. Synthesis of 2-Thioxo-1*H*-pyrazolo[1,5-*a*][1,3,5]triazin-4-one (**3a**)

In a microwave vial, ethoxycarbonyl isothiocyanate (1.0 equiv.) was added drop-wise to a solution of 5-aminopyrazole **1a**–**n** (1.0 equiv.) in dry THF (3 mL) at 0 °C. After complete addition, the mixture was stirred 2 min at room temperature and the vial was sealed, deposited in a microwave reactor and heated at 100 °C for 5 min. After cooling, NaOH 2N (2.0 equiv.) was added and the vessel was sealed again and irradiated at 80 °C for 3 min. After cooling, the resulting aqueous solution was acidified (pH < 5) with HCl 2N then the precipitate was filtered off, washed with water up to neutral pH, triturated with Et_2_O then DCM and dried under vacuum to give **3a** (4.601 g, 94%) as a white solid (4.601 g, 94%) from **1a** (2.5 g, 29.2); m.p. 197–199 °C; IR (neat) ν_max_: 3064.70, 2965.10, 2868.85, 2098.89, 1756.33 (CO), 1719.25, 1637.00, 1530.29, 1422.11, 1363.21, 1236.75, 1173.58, 1131.32 (CS), 1087.90, 1038.44, 961.01, 886.45, 773.52, 699.10, 654.80, 540.74 cm^−1^; ^1^H NMR (300 MHz, DMSO-*d*_6_) δ 5.89 (d, *J* = 1.9 Hz, 1H, H_Ar_), 7.87 (d, *J* = 1.8 Hz, 1H, H_Ar_), 12.74 (s, 1H, NH), 13.45 (s, 1H, NH). ^13^C NMR (75 MHz, DMSO) δ 89.5 (CH_Ar_), 140.6 (C_q_), 141.6 (C_q_), 145.6 (CH_Ar_), 173.0 (C_q_). HRMS (EI-MS): *m*/*z* calcd for C_5_H_4_N_4_SO: 169.0184 [M + H]^+^, found: 169.0190.

#### 3.2.2. General Procedure for the Synthesis of 2-(Methylsulfanyl)pyrazolo[1,5-*a*][1,3,5]triazin-4(3*H*)-ones (**4a**–**n**)

In a microwave vial, ethoxycarbonyl isothiocyanate (1.0 equiv.) was added drop-wise to a solution of 5-aminopyrazole **1a**–**n** (1.0 equiv.) in dry THF (3 mL) at 0 °C. After complete addition, the mixture was stirred 2 min at room temperature and the vial was sealed, deposited in a microwave reactor and heated at 100 °C for 5 min. After cooling, NaOH 2N (2.0 equiv.) was added and the vessel was sealed again and irradiated at 80 °C for 3 min. After cooling, MeI (1.0 equiv.) was added dropwise and the mixture stirred at room temperature for 15 min. The solvent was partially concentrated and the resulting aqueous solution was acidified (pH < 5) with HCl 2N or acetic acid (for **4n** only) and poured in cold water (10 mL). The precipitate was filtered off, washed with water up to neutral pH and the resulting solid was dried under vacuum (**4a**–**n**).

2-(Methylsulfanyl)pyrazolo[1,5-*a*][1,3,5]triazin-4(3*H*)-one (**4a**). White solid (0.325 g, 74%) from **1a** (0.2 g, 2.41 mmol) according to the general procedure; m.p. 244–246 °C; IR (neat) ν_max_: 3097, 3017, 2933, 2904, 2817, 1715 (CO), 1592, 1426, 1337, 1228, 1186, 1156, 1117, 969, 904, 813, 744, 669, 629, 600, 543 cm^−1^; ^1^H NMR (300 MHz, DMSO-*d*_6_) δ 2.53 (s, 3H, CH_3_), 6.35 (d, *J* = 1.9 Hz, 1H, H_Ar_), 7.97 (d, *J* = 1.9 Hz, 1H, H_Ar_), 12.89 (s, 1H, NH); ^13^C NMR (75 MHz, DMSO-*d*_6_) δ 13.1 (CH_3_), 97.1 (CH_Ar_), 143.5 (C_q_), 145.7 (CH_Ar_), 148.4 (C_q_), 157.0 (C_q_); HRMS (EI-MS): *m*/*z* calcd for C_6_H_6_N_4_OS: 183.0335 [M + H]^+^, found: 183.0333.

8-Bromo-2-(methylsulfanyl)pyrazolo[1,5-*a*][1,3,5]triazin-4(3*H*)-one (**4b**). Beige solid (0.530 g, 69%) from **1b** (0.5 g, 2.93 mmol) according to the general procedure; m.p. 268–270 °C; IR (neat) ν_max_: 3123, 3065, 2889, 2824, 1847, 1790 (CO), 1587, 1552, 1480, 1424, 1357, 1317, 1232, 1162, 1000, 898, 768, 736, 672, 651, 625, 543 cm^−1^; ^1^H NMR (300 MHz, DMSO-*d*_6_) δ 2.57 (s, 3H, CH_3_), 8.11 (s, 1H, H_Ar_), 13.07 (s, 1H, OH); ^13^C NMR (75 MHz, DMSO-*d*_6_) δ 13.1 (CH_3_), 83.8 (C_q_), 143.0 (C_q_), 145.5 (CH_Ar_), 145.7 (C_q_), 158.7 (C_q_); HRMS (EI-MS): *m*/*z* calcd for C_6_H_5_BrN_4_OS: 260.9440 [M + H]^+^, found: 260.9453.

2-(Methylsulfanyl)-4-oxo-3,4-dihydropyrazolo[1,5-*a*][1,3,5]triazine-8-carbonitrile (**4c**). White solid (0.090 g, 23%) from **1c** (0.2 g, 1.85 mmol) according to the general procedure; m.p. 254–256 °C; IR (neat) ν_max_: 3128, 3082, 2936, 2235 (CN), 1765 (CO), 1584, 1545, 1485, 1455, 1344, 1226, 1168, 1044, 897, 743, 703, 667, 629, 557 cm^−1^; ^1^H NMR (400 MHz, DMSO-*d*_6_) δ 2.60 (s, 3H, SCH_3_), 8.43 (s, 1H, H_Ar_), 13.16 (br, 1H, OH). ^13^C NMR (101 MHz, DMSO-*d*_6_) δ 13.2 (CH_3_), 81.5 (C_q_), 112.8 (C_q_), 142.8 (C_q_), 146.0 (CH_Ar_), 152.6 (C_q_), 163.3 (C_q_). HRMS (EI-MS): *m*/*z* calcd for C_7_H_5_N_5_OS: 208.0288 [M + H]^+^, found: 208.0290.

Ethyl 2-(methylsulfanyl)-4-oxo-3,4-dihydropyrazolo[1,5-*a*][1,3,5]triazine-8-carboxylate (**4d**). White solid (0.110 g, 34%) from **1d** (0.2 g, 1.29 mmol) according to the general procedure; m.p. 220–222 °C; IR (neat) ν_max_: 3668, 2986, 2901, 1802 (CO_amide_), 1745, 1693 (CO_ester_), 1574, 1537, 1465, 1408, 1382, 1255, 1224, 1173, 1118, 1038, 840, 775, 690, 666, 632, 556 cm^−1^; ^1^H NMR (400 MHz, DMSO-*d*_6_) δ 1.29 (t, *J* = 7.0 Hz, 3H, CH_2_-CH_3_), 2.61 (s, 3H, SCH_3_), 4.23 (q, *J* = 7.0 Hz, 2H, CH_2_-CH_3_), 8.26 (s, 1H, H_Ar_), 13.09 (br, 1H, OH). ^13^C NMR (101 MHz, DMSO-*d*_6_) δ 13.0 (SCH_3_), 14.2 (CH_2_-CH_3_), 59.7 (CH_2_-CH_3_), 102.6 (C_q_), 143.2 (C_q_), 146.0 (CH_Ar_), 148.9 (C_q_), 161.4 (C_q_), 161.4 (C_q_). HRMS (EI-MS): *m*/*z* calcd for C_9_H_10_N_4_O_3_S: 255.0546 [M + H]^+^, found: 255.0540.

8-Methyl-2-(methylsulfanyl)pyrazolo[1,5-*a*][1,3,5]triazin-4(3*H*)-one (**4e**). Black solid (0.510 g, 52%) obtained from **1e** (0.5 g, 5.04 mmol) according to the general procedure, m.p. 186–188 °C; IR (neat) ν_max_: 3674, 2987, 2900, 1758 (CO), 1606, 1558, 1487, 1439, 1347, 1239, 1173, 1053, 981, 899, 821, 738, 724, 661, 628, 519 cm^−1^; ^1^H NMR (400 MHz, DMSO-*d*_6_) δ 2.09 (s, 3H, CH_3_), 2.56 (s, 3H, SCH_3_), 7.86 (s, 1H, H_Ar_), 12.70 (s, 1H, OH). ^13^C NMR (101 MHz, DMSO-*d*_6_) δ 7.1 (CH_3_), 12.9 (SCH_3_), 105.5 (C_q_), 143.6 (C_q_), 145.2 (C_q_), 146.6 (CH_Ar_), 155.3 (C_q_). HRMS (EI-MS): *m*/*z* calcd for C_7_H_8_N_4_OS: 197.0419 [M + H]^+^, found: 197.0496.

8-Ethyl-2-(methylsulfanyl)pyrazolo[1,5-*a*][1,3,5]triazin-4(3*H*)-one (**4f**). White solid (0.655 g, 65%) obtained from **1f** (0.5 g, 4.41 mmol) according to the general procedure; m.p. 188–190 °C; IR (neat) ν_max_: 3498, 3080, 2961, 2873, 2634, 1993, 1761 (CO), 1598, 1563, 1497, 1450, 1353, 1238, 1171, 1065, 1033, 960, 908, 823, 740, 703, 663, 630, 547 cm^−1^; ^1^H NMR (300 MHz, DMSO-*d*_6_) δ 1.20 (t, *J* = 7.6 Hz, 3H, CH_2_-CH_3_), 2.52–2.60 (m, 5H, CH_2_-CH_3_ and SCH_3_), 7.91 (s, 1H, H_Ar_), 12.73 (s, 1H, OH). ^13^C NMR (75 MHz, DMSO-*d*_6_) δ 12.9 (SCH_3_), 14.4 (CH_2_-CH_3_), 15.5 (CH_2_-CH_3_), 112.0 (C_q_), 143.7 (C_q_), 144.7 (C_q_), 145.5 (CH_Ar_), 155.3 (C_q_); HRMS (EI-MS): *m*/*z* calcd for C_8_H_10_N_4_OS: 211.0648 [M + H]^+^, found: 211.0655.

8-Isopropyl-2-(methylsulfanyl)pyrazolo[1,5-*a*][1,3,5]triazin-4(3*H*)-one (**4g**). Pale yellow solid (0.964 g, 75%) obtained from **1g** (0.750 g, 5.69 mmol) according to the general procedure; m.p. 203–205 °C; IR (neat) ν_max_: 3079, 2953, 2925, 2865, 2798, 2634, 1759 (CO), 1595, 1572, 1554, 1492, 1448, 1359, 1312, 1242, 1175, 1030, 908, 826, 741, 701, 665, 629, 553 cm^−1^; ^1^H NMR (400 MHz, DMSO-*d*_6_) δ 1.25 (d, *J* = 7.0 Hz, 6H, 2 × CH_3_-_iPr_), 2.54 (s, 3H, SCH_3_), 3.01 (hept, *J* = 7.0 Hz, 1H, H_iPr_), 7.91 (s, 1H), 12.73 (s, 1H, OH). ^13^C NMR (101 MHz, DMSO-*d*_6_) δ 12.9 (SCH_3_), 22.9 (2 × CH_3_-_iPr_), 23.1 (CHiPr), 116.9 (C_q_), 143.6 (C_q_), 144.1 (C_q_), 144.2 (CH_Ar_), 155.0 (C_q_). HRMS (EI-MS): *m*/*z* calcd for C_9_H_12_N_4_OS: 225.0805 [M + H]^+^, found: 225.0806.

8-Cyclopropyl-2-(methylsulfanyl)pyrazolo[1,5-*a*][1,3,5]triazin-4(3*H*)-one (**4h**). Beige solid (0.410 g, 60%) obtained from **1h** (0.380 g, 3.08 mmol) according to the general procedure; m.p. 225–227 °C; IR (neat) ν_max_: 3079, 2995, 2883, 2635, 1758 (CO), 1606, 1561, 1504, 1449, 1349, 1313, 1238, 1207, 1167, 1030, 906, 876, 813, 739, 696, 660, 627, 548 cm^−1^; ^1^H NMR (300 MHz, DMSO-*d*_6_) δ 0.77–0.93 (m, 4H, 2 × CH_2-cPr_), 1.83 (ddd, *J* = 16.8, 8.3, 5.2 Hz, 1H, CH_cPr_), 2.54 (s, 3H, CH_3_), 7.79 (s, 1H, H_Ar_), 12.70 (s, 1H, OH). ^13^C NMR (75 MHz, DMSO-*d*_6_) δ 4.8 (CH_cPr_), 7.2 (2 × CH_2-cPr_), 12.9 (CH_3_), 112.7 (C_q_), 143.5 (C_q_), 144.1 (CH_Ar_), 144.6 (C_q_), 155.1 (C_q_). HRMS (EI-MS): *m*/*z* calcd for C_9_H_10_N_4_OS: 223.0348 [M + H]^+^, found: 223.0661.

8-Cyclobutyl-2-(methylsulfanyl)pyrazolo[1,5-*a*][1,3,5]triazin-4(3*H*)-one (**4i**). White solid (0.190 g, 55%) obtained from **1i** (0.2 g, 1.46 mmol) according to the general procedure; m.p. 222–224 °C; IR (neat) ν_max_: 3667, 3080, 2969, 2900, 2634, 2119, 1759 (CO), 1596, 1567, 1497, 1454, 1348, 1240, 1197, 1163, 1053, 988, 906, 822, 739, 665, 629, 546 cm^−1^; ^1^H NMR (400 MHz, DMSO-*d*_6_) δ 1.81–2.03 (m, 4H, CH_2_-CH_2_-CH), 2.25 (qd, *J* = 8.5, 2.7 Hz, 4H, CH_2_-CH_2_-CH), 2.55 (s, 3H, SCH_3_), 3.54 (p, *J* = 8.7 Hz, 1H, H_cBut_), 8.00 (s, 1H, H_Ar_), 12.72 (s, 1H, OH). ^13^C NMR (101 MHz, DMSO-*d*_6_) δ 12.9 (SCH_3_), 18.4 (2 × CH_2_-CH_2_-CH), 29.0 (CH_cBut_), 29.5 (2 × CH_2_-CH_2_-CH), 114.8 (C_q_), 143.6 (C_q_), 144.2 (C_q_), 144.9 (CH_Ar_), 155.3 (C_q_). HRMS (EI-MS): *m*/*z* calcd for C_10_H_12_N_4_OS: 237.0805 [M + H]^+^, found: 237.0809.

2-(Methylsulfanyl)-8-phenyl-pyrazolo[1,5-*a*][1,3,5]triazin-4(3*H*)-one (**4j**). White solid (0.500 g, 65%) obtained from **1j** (0.5 g, 2.98 mmol) according to the general procedure; m.p. 277–279 °C; IR (neat) ν_max_: 3491, 3065, 2890, 2824, 1944, 1756 (CO), 1594, 1559, 1460, 1432, 1356, 1228, 1173, 1145, 1094, 981, 934, 904, 795, 755, 713, 687, 670, 628, 550, 511 cm^−1^; ^1^H NMR (400 MHz, DMSO-*d*_6_) δ 2.64 (s, 3H, SCH_3_), 7.25 (t, *J* = 7.4 Hz, 1H, H_Ar_), 7.42 (t, *J* = 7.6 Hz, 2H, 2 × H_Ar_), 8.03 (d, *J* = 7.7 Hz, 2H, 2 × H_Ar_), 8.53 (s, 1H, H_Ar_), 12.98 (s, 1H, OH). ^13^C NMR (101 MHz, DMSO-*d*_6_) δ 13.2 (SCH_3_), 109.8 (C_q_), 125.7 (2 × CH_Ar_), 126.4 (CH_Ar_), 128.7 (2 × CHAr), 131.2 (C_q_), 143.5 (C_q_), 143.8 (CH_Ar_), 144.1 (C_q_), 157.6 (C_q_). HRMS (EI-MS): *m*/*z* calcd for C_12_H_10_N_4_OS: 259.0648 [M + H]^+^, found: 259.0657.

2-(Methylsulfanyl)-8-(*p*-tolyl)pyrazolo[1,5-*a*][1,3,5]triazin-4(3*H*)-one (**4k**). White solid (0.560 g, 68%) obtained from **1k** (0.550 g, 3.02 mmol) according to the general procedure; m.p. > 290 °C; IR (neat) ν_max_: 3486, 3064, 3007, 2884, 1759 (CO), 1595, 1561, 1511, 1455, 1407, 1352, 1316, 1255, 1228, 1172, 1144, 982, 907, 809, 741, 695, 671, 626, 550, 514 cm^−1^; ^1^H NMR (300 MHz, DMSO-*d*_6_) δ 2.31 (s, 3H, SCH_3_), 2.63 (s, 3H, CH_3_-Ph), 7.23 (d, *J* = 7.8 Hz, 2H, 2 × H_Ar_), 7.91 (d, *J* = 8.0 Hz, 2H, 2 × H_Ar_), 8.48 (s, 1H, H_Ar_), 12.95 (s, 1H, OH). ^13^C NMR (75 MHz, DMSO-*d*_6_) δ 13.2 (SCH_3_), 20.8 (CH_3_-Ph), 109.9 (C_q_), 125.6 (2 × CH_Ar_), 128.2 (C_q_), 129.3 (2 × CH_Ar_), 135.5 (C_q_), 143.5 (C_q_), 143.7 (CH_Ar_), 143.8 (C_q_), 157.2 (C_q_). HRMS (EI-MS): *m*/*z* calcd for C_13_H_12_N_4_OS: 273.0805 [M + H]^+^, found: 273.0810.

8-(4-Chlorophenyl)-2-(methylsulfanyl)pyrazolo[1,5-*a*][1,3,5]triazin-4(3*H*)-one (**4l**). White solid (0.255 g, 84%) obtained from **1l** (0.2 g, 1.03 mmol) according to the general procedure; m.p. > 290 °C; IR (neat) ν_max_: 3674, 2987, 2900, 2123, 1753 (CO), 1595, 1560, 1511, 1453, 1352, 1303, 1247, 1173, 1038, 979, 906, 815, 742, 694, 671, 625, 589, 551, 522 cm^−1^; ^1^H NMR (400 MHz, DMSO-*d*_6_) δ 2.64 (s, 3H, SCH_3_), 7.48 (d, *J* = 8.3 Hz, 2H, 2 × H_Ar_), 8.06 (d, *J* = 8.4 Hz, 2H, 2 × H_Ar_), 8.54 (s, 1H, H_Ar_), 13.05 (s, 1H, OH) ^13^C NMR (101 MHz, DMSO-*d*_6_) δ 13.3 (SCH_3_), 108.6 (C_q_), 127.2 (2 × CH_Ar_), 128.7 (2 × CH_Ar_), 130.2 (C_q_), 130.7 (C_q_), 143.4 (C_q_), 143.6 (CH_Ar_), 144.3 (C_q_), 158.1 (C_q_). HRMS (EI-MS): *m*/*z* calcd for C12H9ClN4OS: 293.0258 [M + H]^+^, found: 293.0263.

8-(4-Methoxyphenyl)-2-(methylsulfanyl)pyrazolo[1,5-*a*][1,3,5]triazin-4(3*H*)-one (**4m**). White solid (0.510 g, 64%) obtained from **1m** (0.525 g, 2.77 mmol) according to the general procedure; m.p. > 290 °C; IR (neat) ν_max_: 3062, 2883, 1759 (CO), 1587, 1553, 1487, 1454, 1401, 1351, 1224, 1174, 1086, 983, 906, 808, 740, 676, 663, 627, 552, 511 cm^−1^; ^1^H NMR (400 MHz, DMSO-*d*_6_) δ 2.63 (s, 3H, SCH_3_), 3.78 (s, 3H, OCH_3_), 6.84–7.17 (m, 2H, 2 × H_Ar_), 7.88–8.12 (m, 2H, 2 × H_Ar_), 8.46 (s, 1H, H_Ar_), 12.92 (s, 1H, OH). ^13^C NMR (101 MHz, DMSO-*d*_6_) δ 13.2 (SCH_3_), 55.1 (OCH_3_), 109.7 (C_q_), 114.2 (2 × CH_Ar_), 123.6 (C_q_), 126.9 (2 × CH_Ar_), 143.3 (C_q_), 143.5 (CH_Ar_), 143.5 (C_q_), 156.9 (C_q_), 157.9 (C_q_). HRMS (EI-MS): *m*/*z* calcd for C_13_H_12_N_4_O_2_S: 289.0754 [M + H]^+^, found: 289.0752.

2-(Methylsulfanyl)-8-(2-pyridyl)pyrazolo[1,5-*a*][1,3,5]triazin-4(3*H*)-one (**4n**). White solid (0.280 g, 86%) obtained from **1n** (0.2 g, 1.25 mmol) according to the general procedure; m.p. 271–273 °C; IR (neat) ν_max_: 3662, 3395, 3098, 2987, 1631 (CO), 1573, 1474, 1454, 1425, 1368, 1289, 1253, 1196, 1144, 1048, 991, 932, 776, 642, 515 cm^−1^; ^1^H NMR (400 MHz, DMSO-*d*_6_) δ 2.66 (s, 3H, SCH_3_), 7.24 (dd, *J* = 7.5, 4.9 Hz, 1H, H_Ar_), 7.86 (t, *J* = 7.9 Hz, 1H, H_Ar_), 8.28 (d, *J* = 8.0 Hz, 1H, H_Ar_), 8.51 (s, 1H, H_Ar_), 8.57 (d, *J* = 4.9 Hz, 1H, H_Ar_), 13.11 (s, 1H, OH). ^13^C NMR (101 MHz, DMSO-*d*_6_) δ 13.4 (SCH_3_), 110.3 (C_q_), 120.5 (CH_Ar_), 121.4 (CH_Ar_), 137.0 (CH_Ar_), 143.5 (C_q_), 144.2 (CH_Ar_), 145.4 (C_q_), 149.1 (C_q_), 150.2 (C_q_), 159.3 (C_q_). HRMS (EI-MS): *m*/*z* calcd for C_11_H_9_N_5_OS: 260.0601 [M + H]^+^, found: 260.0603.

#### 3.2.3. Synthesis of Pyrazolo[1,5-*a*][1,3,5]triazines **5** and **6**

4-Chloro-2-(methylsulfanyl)pyrazolo[1,5-*a*][1,3,5]triazine (**5**) in a 250 mL sealed flask with a stir bar were charged, **4a** (6.0 g, 32.9 mmol, 1.0 eq.), POCl_3_ (31.10 mL, 329.3 mmol, 10.0 eq.) and *N*,*N*-dimethylaniline (4.22 mL, 32.9 mmol, 1.0 eq.). The vessel was sealed and heated at 110 °C (heating block) for 3 h. After cooling, the mixture was concentrated and the resulting residue was taken up in dichloromethane (100 mL, DCM). The organic solution was washed with saturated NaHCO_3_ (3 × 100 mL) The aqueous layer (pH > 7) was extracted twice with DCM (3 × 30 mL) and the resulting organic solution was dried over MgSO_4_, filtered and concentrated. Purification by flash chromatography with DCM/petroleum ether (5/5 to 7/3) as eluent gave **5** (6.20 g, 94%) as a yellow solid; m.p. 143–145 °C; IR (neat) ν_max_: 3675, 3140, 2987, 2912, 2901, 2114, 1991, 1822, 1732, 1650, 1595, 1512, 1464, 1418, 1359, 1315, 1223, 1185, 1124, 1005, 896, 862, 773, 654, 630, 601, 535 cm^−1^; ^1^H NMR (300 MHz, DMSO-*d*_6_) δ 2.60 (s, 3H, SCH_3_), 6.50 (d, *J* = 2.2 Hz, 1H, H_Ar_), 8.15 (d, *J* = 2.2 Hz, 1H, H_Ar_). HRMS (EI-MS): *m*/*z* calcd for C_6_H_5_ClN_4_S: 200.9996 [M + H]+, found: 201.0002.

4-Chloro-2-(methylsulfanyl)pyrazolo[1,5-*a*][1,3,5]triazine (**6**): a microwave vial (10–20 mL) was charged with a stir bar, **5** (2.0 g, 9.97 mmol, 1.0 eq.) and CHCl_3_ (17 mL) and NBS (2.307 g, 12.96 mmol, 1.3 eq.). The vial was sealed and the resulting mixture was heated at 80 °C for 8 min under microwave irradiation. After cooling, celite was added and the solvent removed for a solid deposit on a flash chromatography, with DCM/petroleum ether (4/6) as eluent. Product **6** (2.641 g, 95%) was obtained as a pale yellow solid; m.p. 150–152 °C; IR (neat) ν_max_: 3674, 2987, 2900, 2123, 1847, 1792, 1768, 1587, 1549, 1529, 1467, 1417, 1357, 1316, 1232, 1157, 1067, 1001, 927, 768, 735, 673, 650, 625, 543 cm^−1^; ^1^H NMR (300 MHz, DMSO-*d*_6_) δ 2.66 (s, 3H, SCH_3_), 8.13 (s, 1H, H_Ar_).

The instability of **6** and **5** in solution do not allowed more complete analysis (e.g., ^13^C NMR, HRMS in the case of **6**) and yield to the starting triazine-4-one **4a**, suggesting fast hydrolysis.

## 4. Conclusions

The innovative conditions described in this work are combining the efficient heating obtained under microwave irradiation and the interest to perform sequential one-pot reactions, discarding the tedious work-up and purification processes of intermediates compounds, saving energy, time and minimizing wastes.

The process described in this study allows reproducible and safe operating conditions for convenient and rapid access to valuable C8-substituted 2-(methylsulfanyl)pyrazolo[1,5-*a*][1,3,5]triazin-4(3*H*)-ones (**4a**–**n**). In addition, multi-gram scale reactions were also successfully performed, confirming the interest of this procedure.

## Data Availability

The data presented in this study are available in supplementary material.

## Supplementary Materials

The following are available online, ^1^H NMR and ^13^C NMR spectra of compounds **4a**–**n** and ^1^H NMR of compounds **5** and **6**.

## Sample Availability

Not available.
