# Supplementary material for: Microwave-Assisted Sequential One-Pot Synthesis of 8-Substituted Pyrazolo[1,5-a][1,3,5]triazines
Source: Molecules. 2021 Jun 10;26(12):3540. doi: 10.3390/molecules26123540 (PMC8230123; doi:10.3390/molecules26123540)

molecules

Supplementary Materials

Article

Microwave-assisted sequential one-pot synthesis of 8-substituted pyrazolo[1,5-*a*][1,3,5]triazines

Jonathan Elie [1,2], Corinne Fruit [1] and Thierry Besson [1,*]

[1] Normandie Univ, UNIROUEN, INSA Rouen, CNRS, COBRA UMR 6014, F-76000 Rouen, France; corinne.fruit@univ-rouen.fr (C.F.)
[2] Perha Pharmaceuticals, Perharidy Peninsula, 29680 Roscoff, France; elie@perha-pharma.com (J.E.)
* Correspondence: thierry.besson@univ-rouen.fr; Tel.: +33 235-522-904

		Page
1.	^{1}H NMR, ^{13}C NMR, DEPY 135, HMBC, HSQC and NOESY Spectrum of products **4a**	S2 to S4
2.	^{1}H NMR and ^{13}C NMR Spectra of products **4b** to **4f**	**S**5 to S9
3.	^{1}H NMR, ^{13}C NMR, DEPY 135, HMBC, HSQC and NOESY Spectrum of products **4g**	S10 to S12
4.	^{1}H NMR and ^{13}C NMR Spectra of products **4h** to **4n**	S5 to S19
5.	^{1}H NMR, ^{13}C NMR and DEPT135 Spectra of products **3a**	S20 to S21
6.	^{1}H NMR Spectra of **5** and **6**	S22

2-(Methylsulfanyl)pyrazolo[1,5-*a*][1,3,5]triazin-4(3*H*)-one (**4a**)

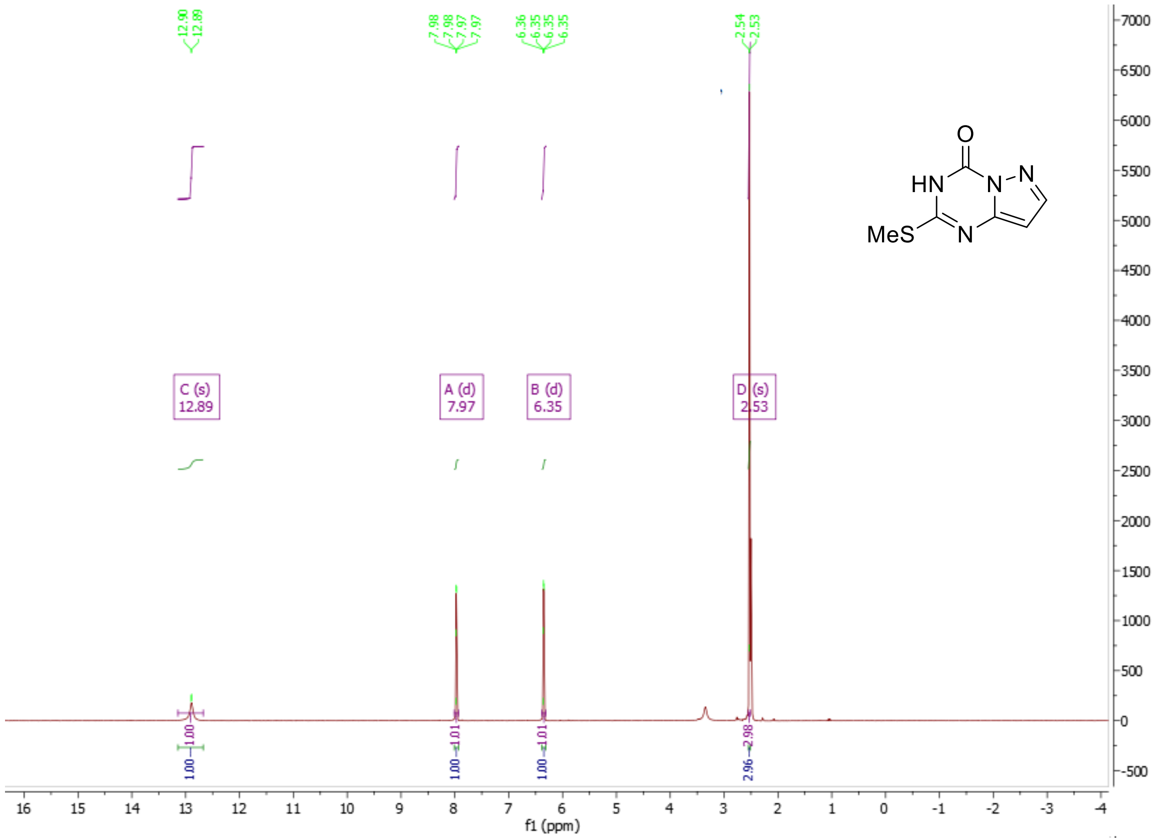

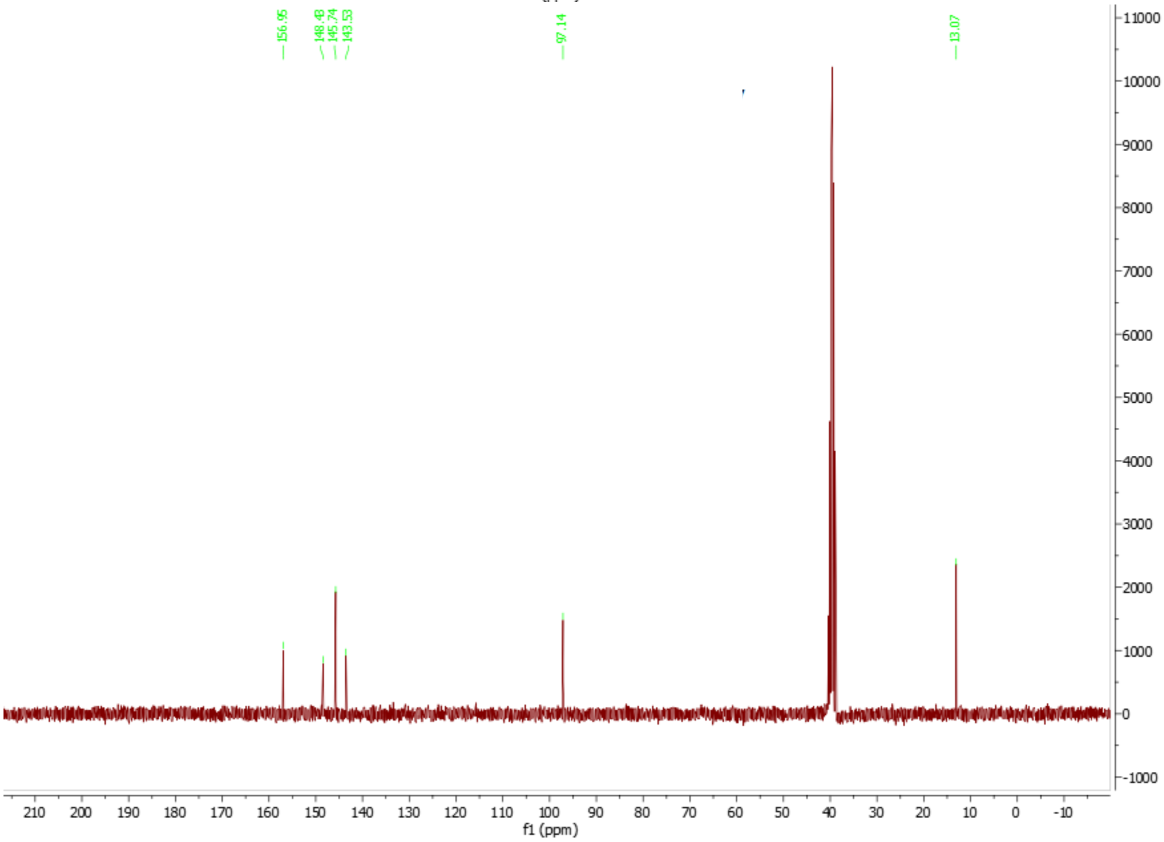

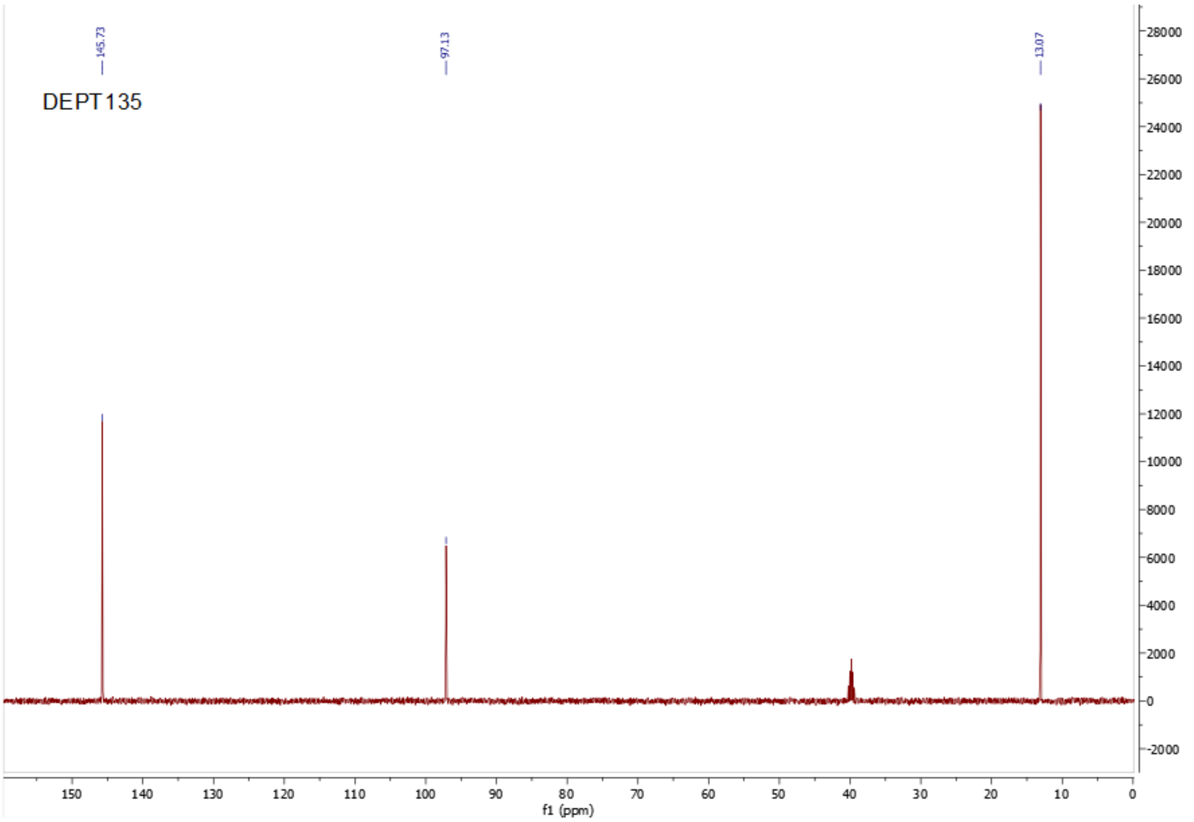
145.73
97.13
13.07
DEPT135
28000
26000
24000
22000
20000
18000
16000
14000
12000
10000
8000
6000
4000
2000
0
-2000
150
140
130
120
110
100
90
80
70
60
50
40
30
20
10
0
f1 (ppm)

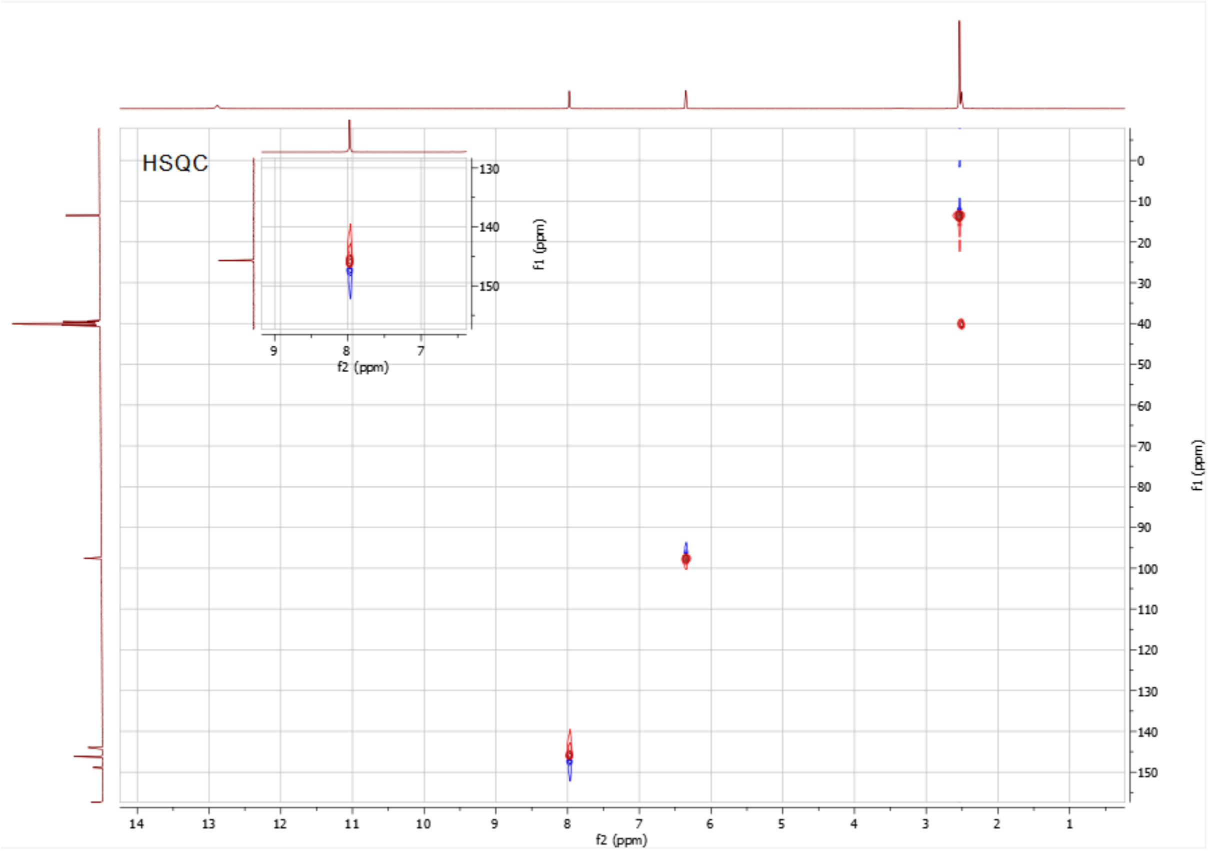
HSQC
130
140
150
9
8
7
f2 (ppm)
f1 (ppm)
0
10
20
30
40
50
60
70
80
90
100
110
120
130
140
150
14
13
12
11
10
9
8
7
6
5
4
3
2
1
f2 (ppm)
f1 (ppm)

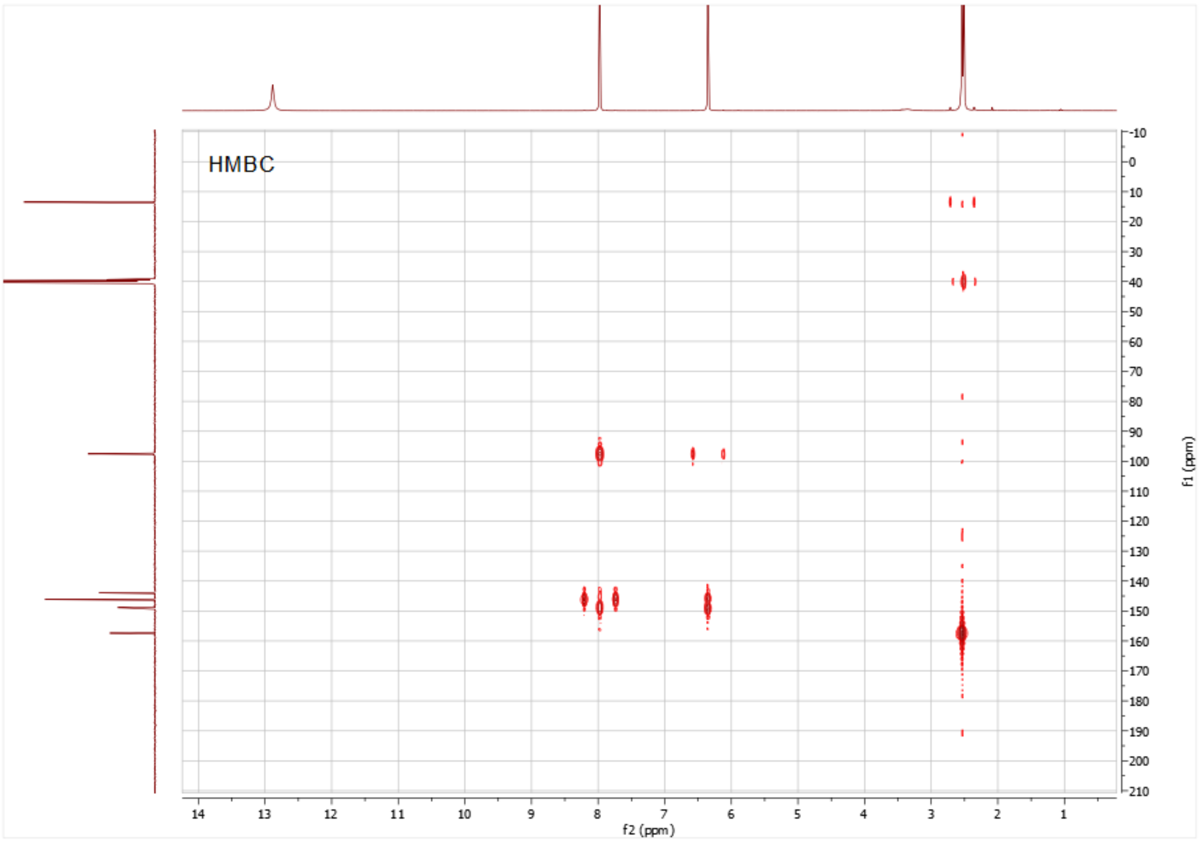
HMBC
f2 (ppm)
f1 (ppm)

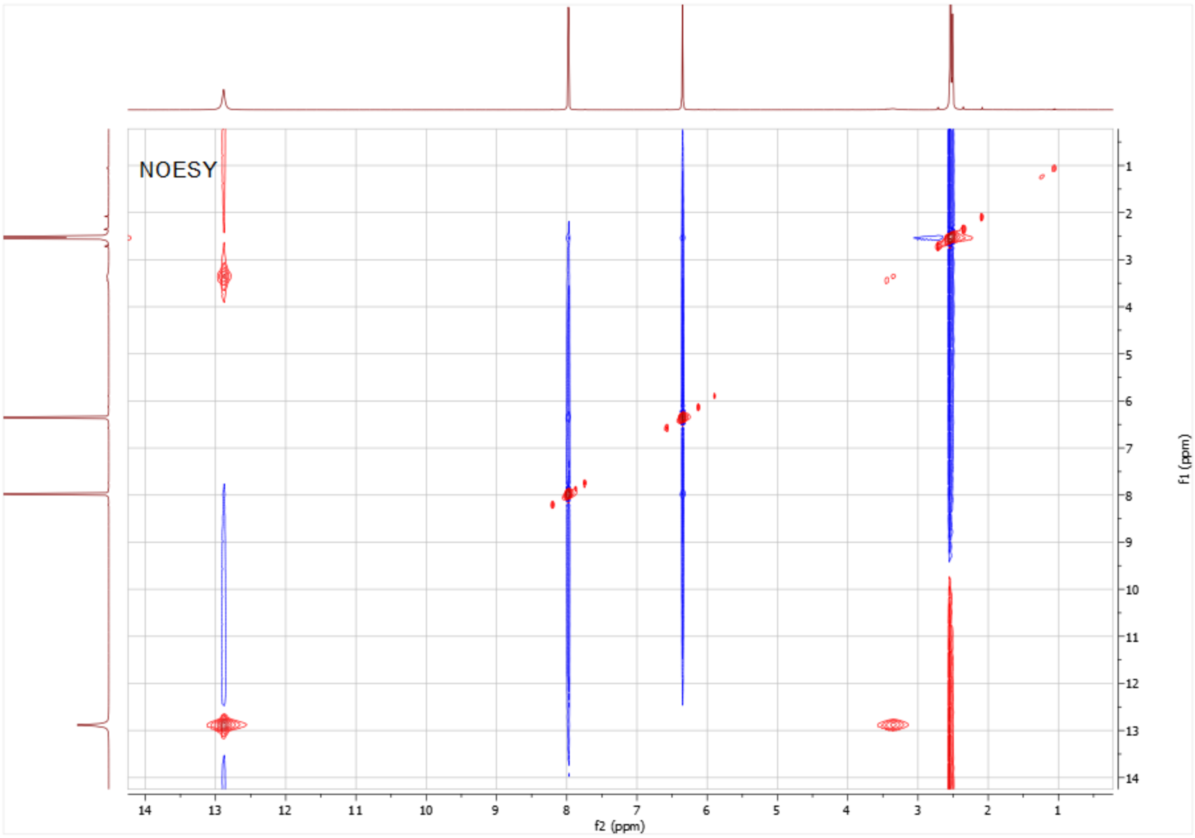
NOESY
f2 (ppm)
f1 (ppm)

8-Bromo-2-(methylsulfanyl)pyrazolo[1,5-a][1,3,5]triazin-4(3H)-one (**4b**)

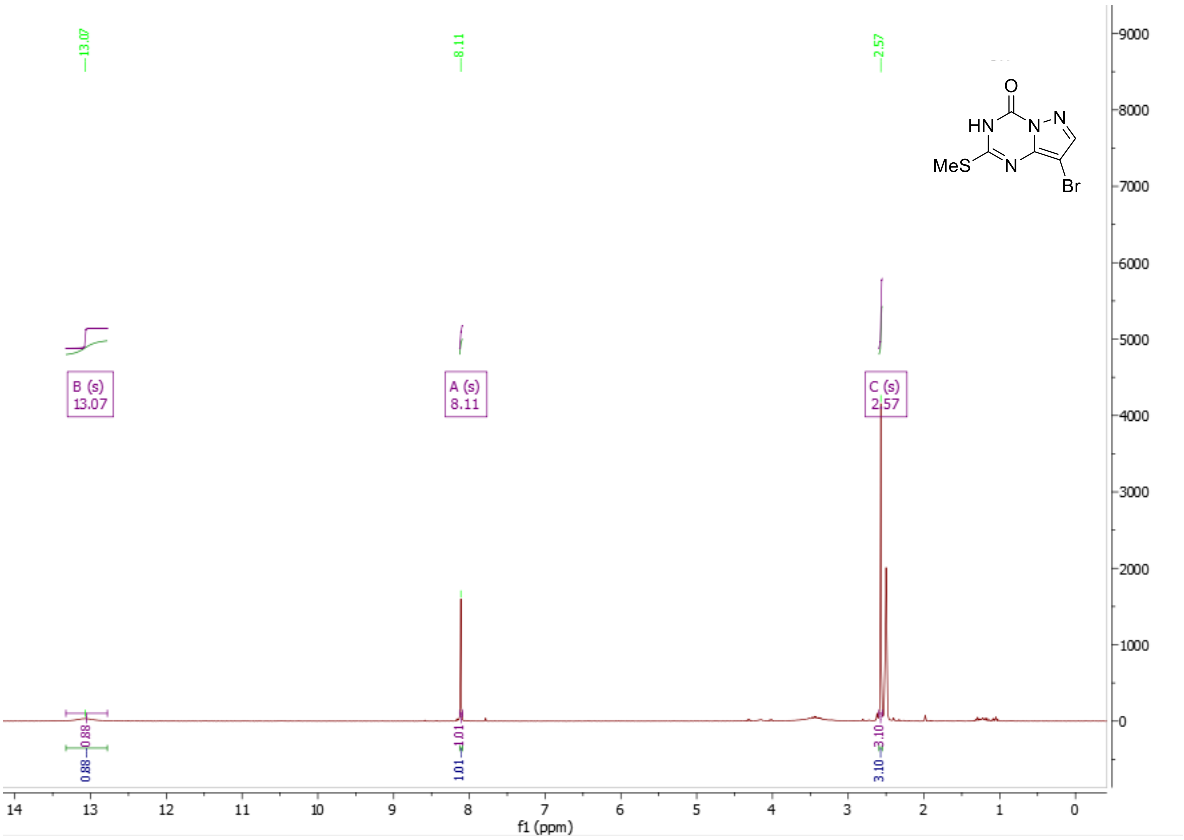

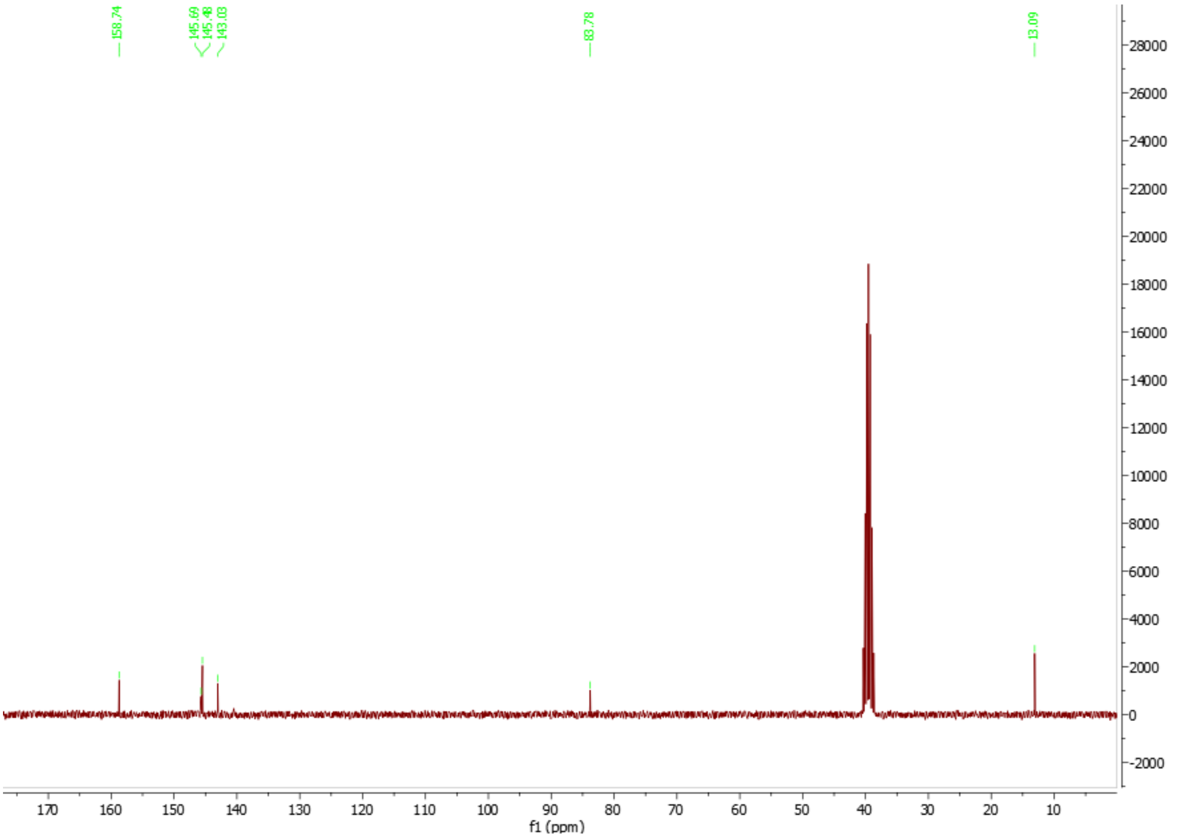

2-(Methylsulfanyl)-4-oxo-3,4-dihydropyrazolo[1,5-a][1,3,5]triazine-8-carbonitrile (**4c**)

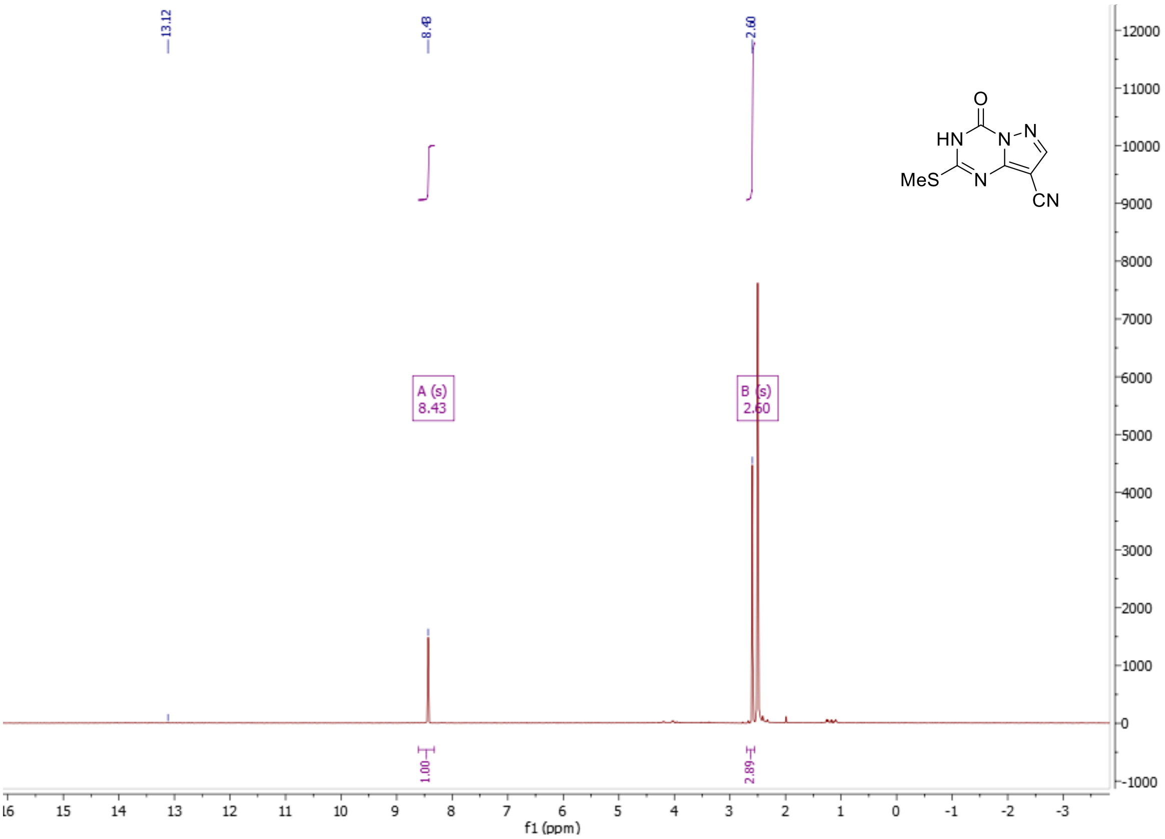

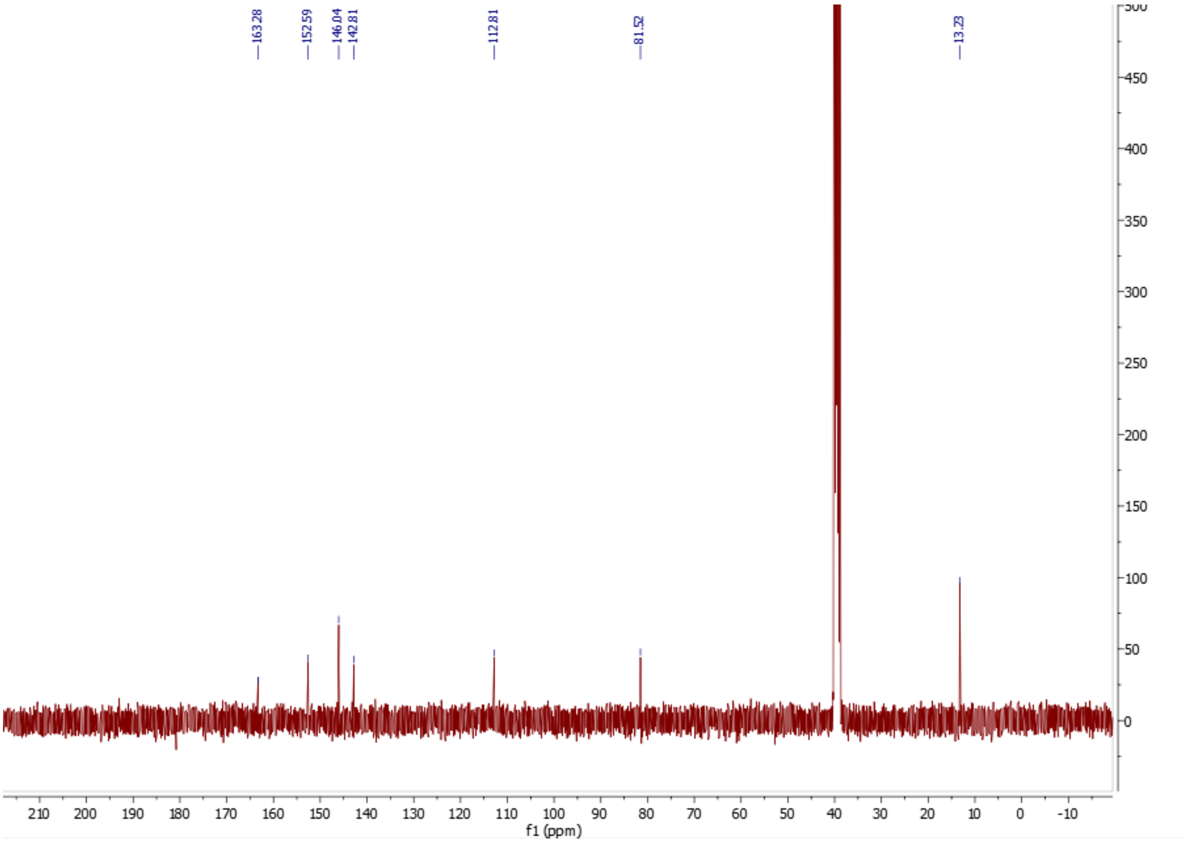

Ethyl 2-(methylsulfanyl)-4-oxo-3,4-dihydropyrazolo[1,5-a][1,3,5]triazine-8-carboxylate (**4d**)

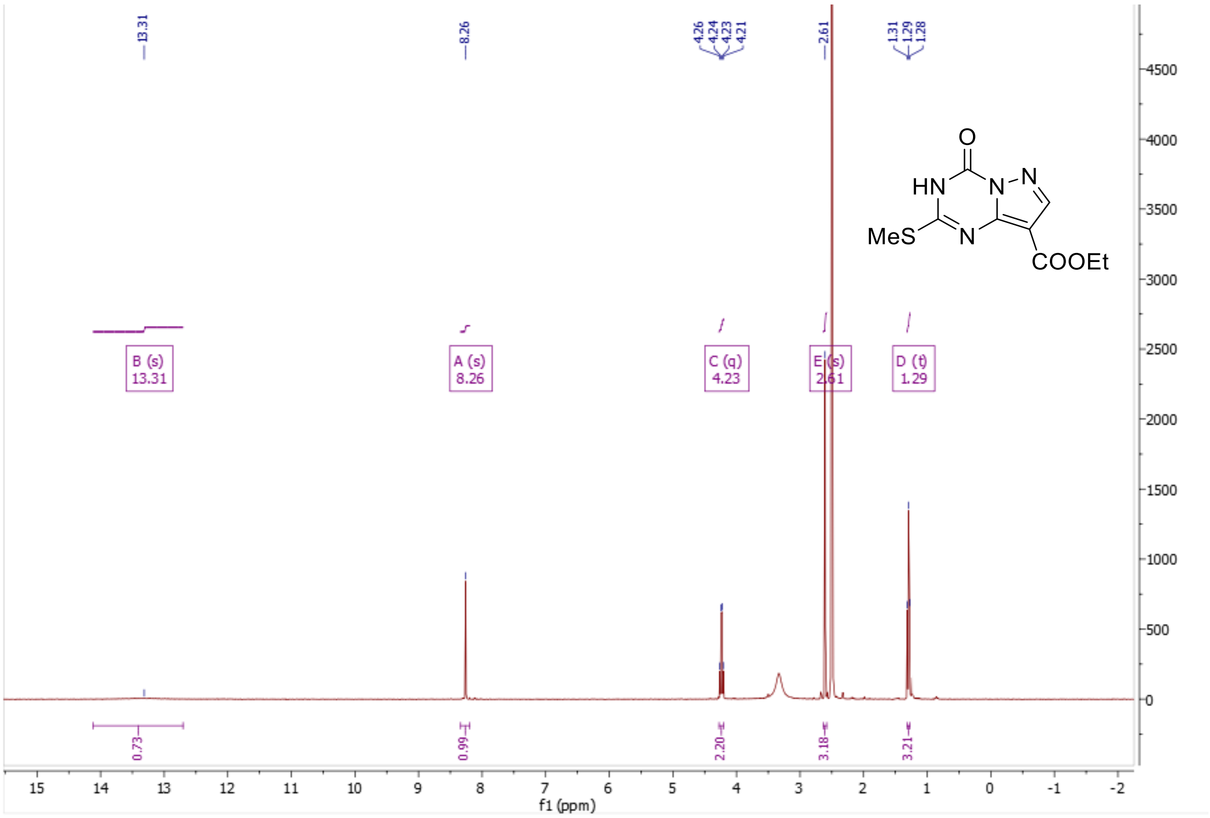

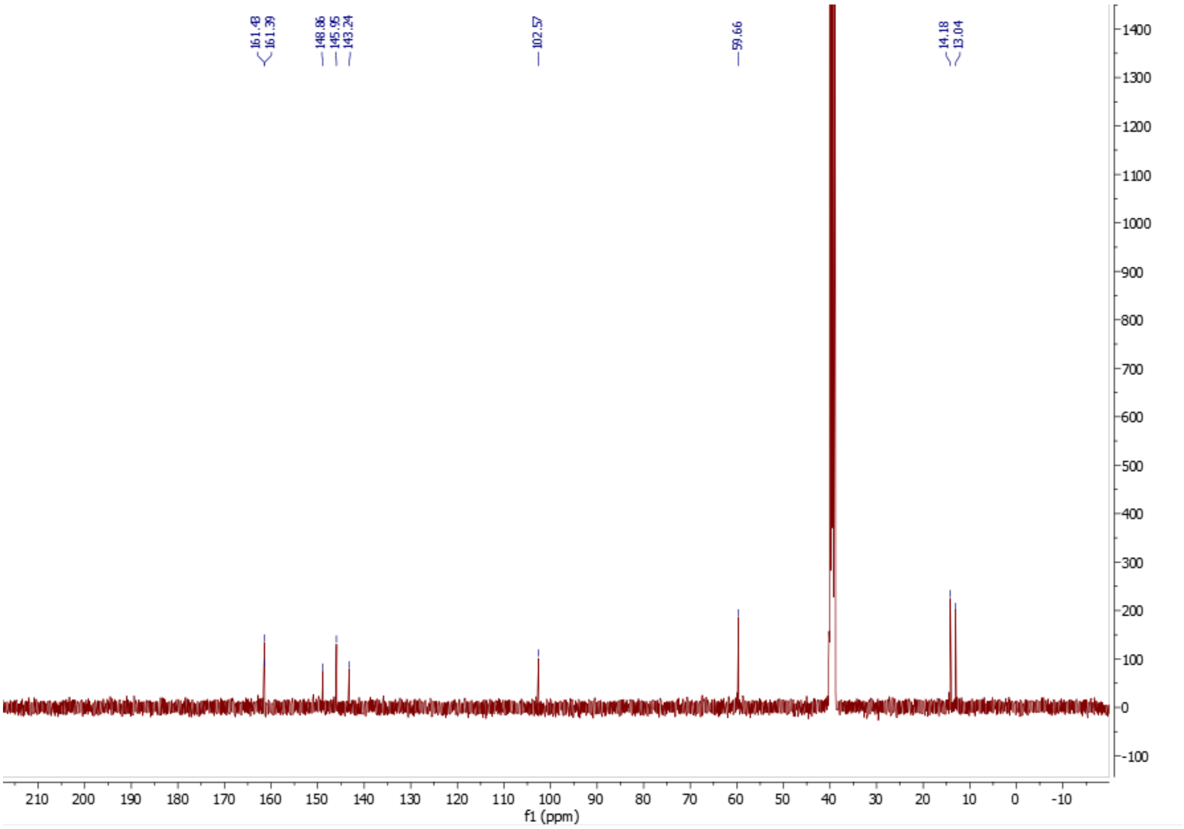

8-Methyl-2-(methylsulfanyl)pyrazolo[1,5-a][1,3,5]triazin-4(3H)-one (**4e**)

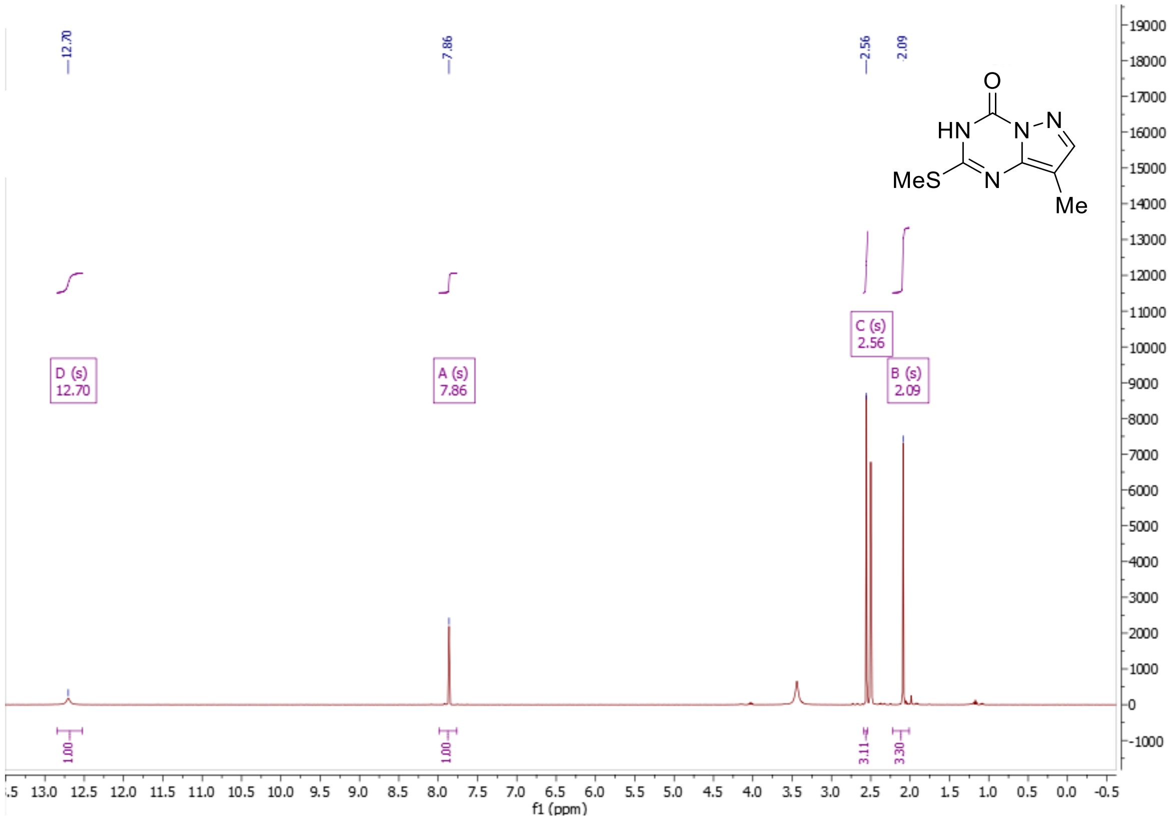

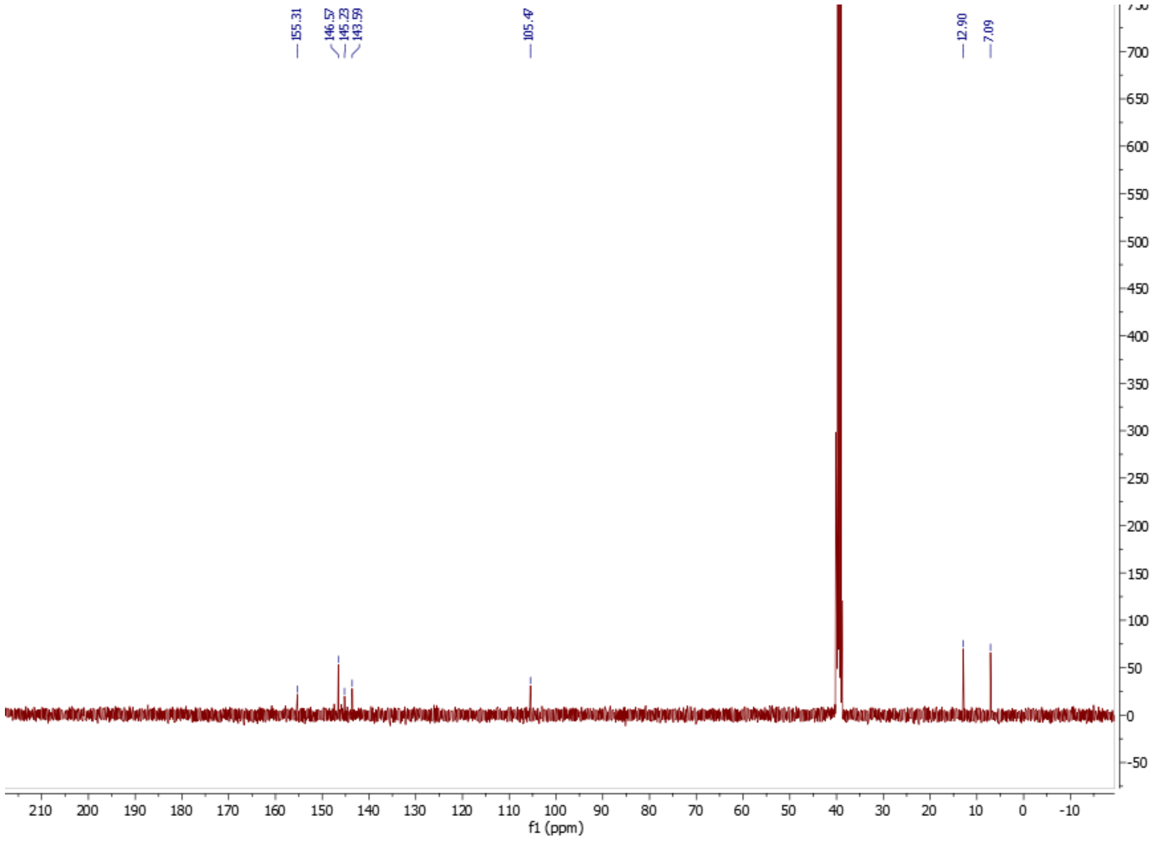

8-Ethyl-2-(methylsulfanyl)pyrazolo[1,5-a][1,3,5]triazin-4(3H)-one (**4f**)

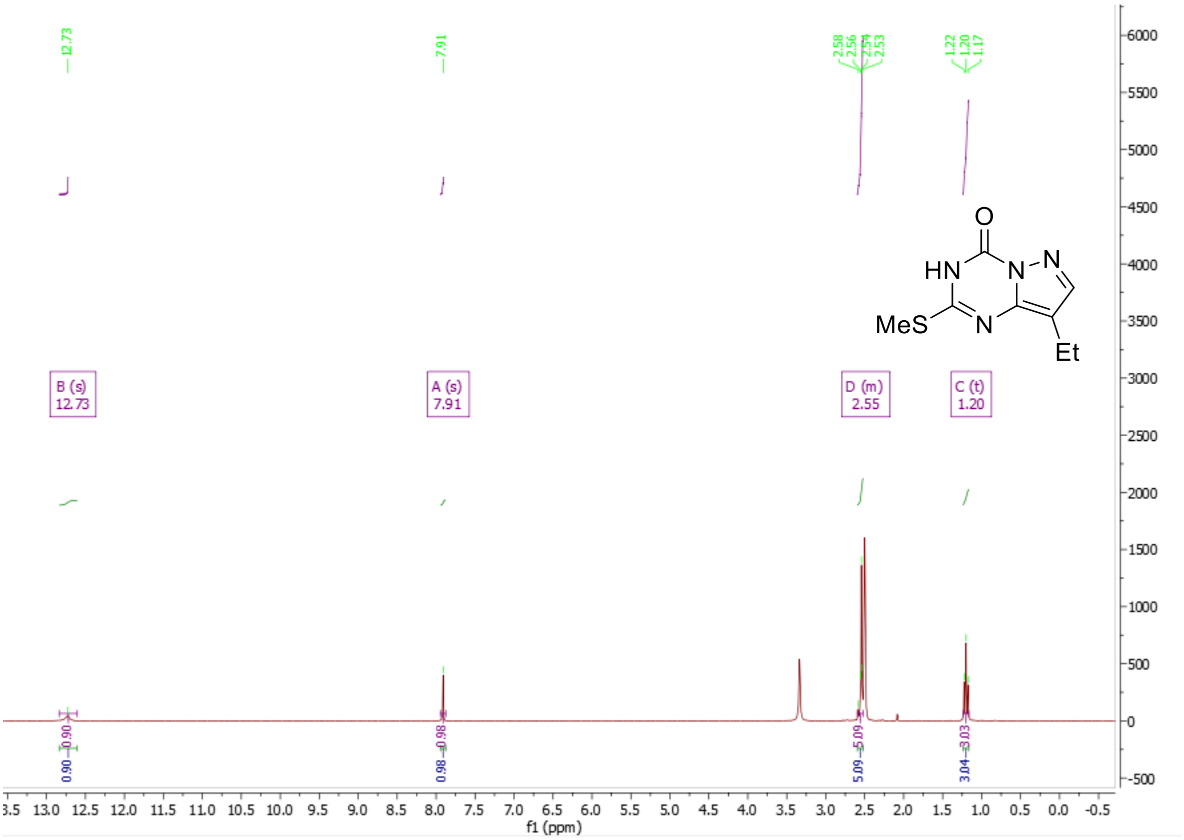

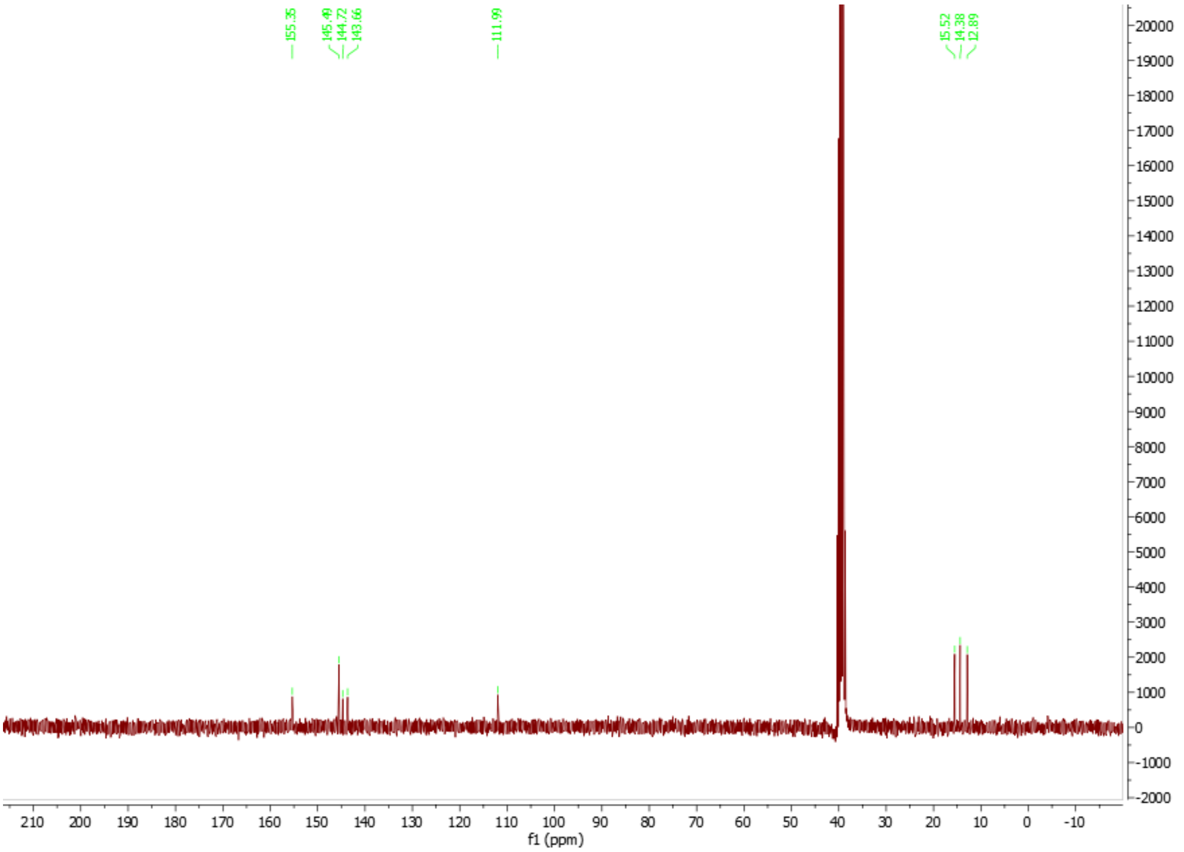

8-Isopropyl-2-(methylsulfanyl)pyrazolo[1,5-a][1,3,5]triazin-4(3H)-one (**4g**)

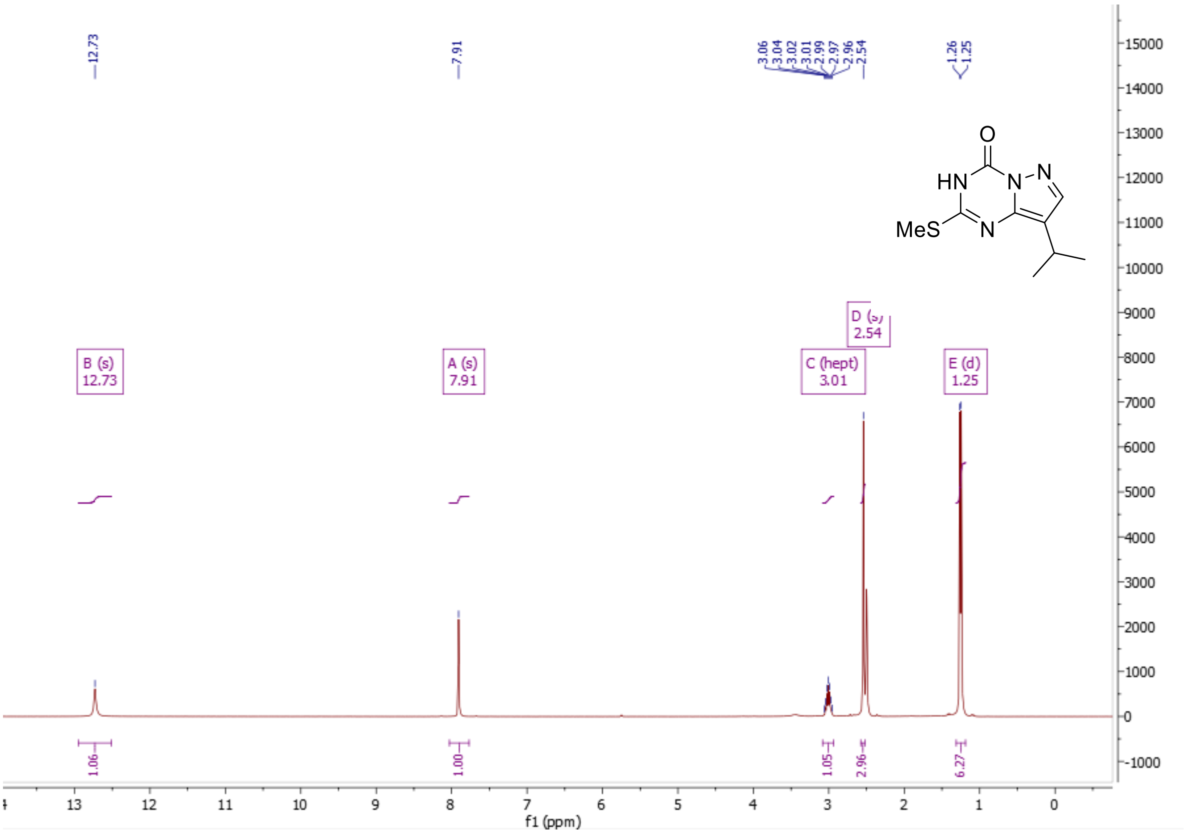

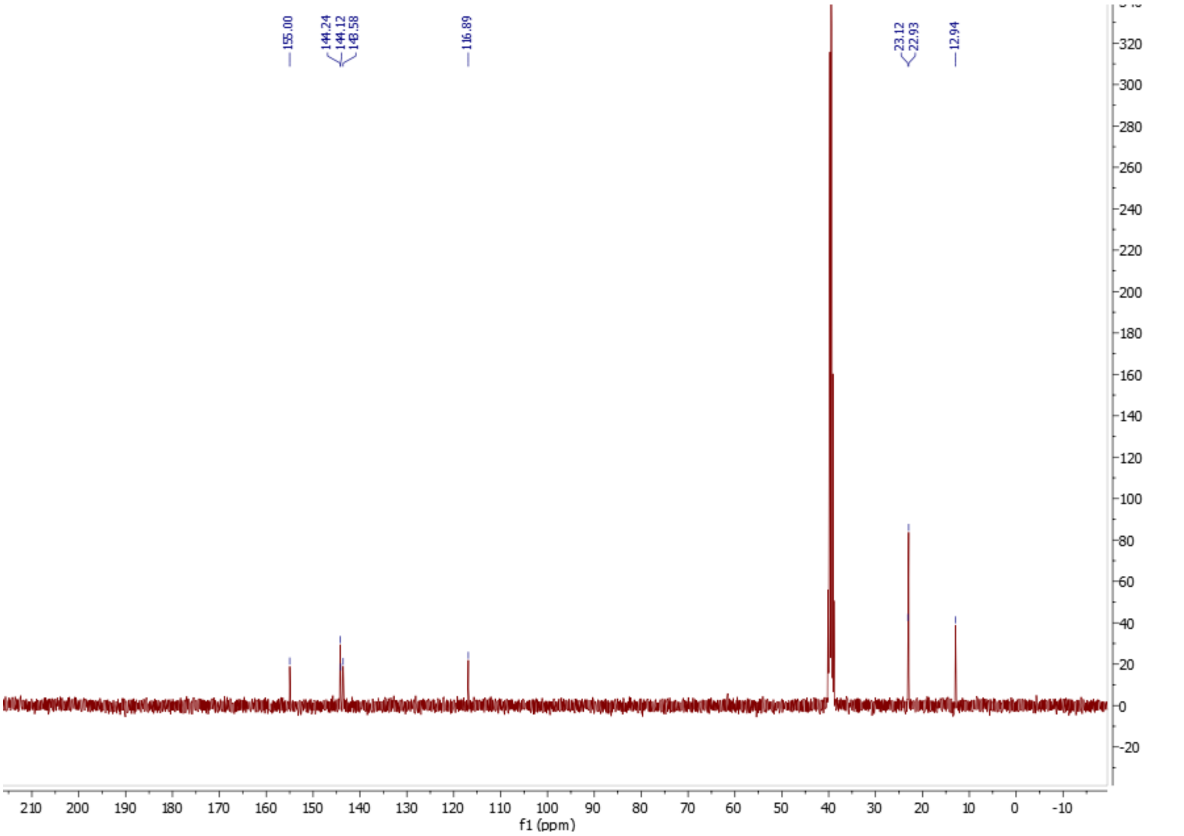

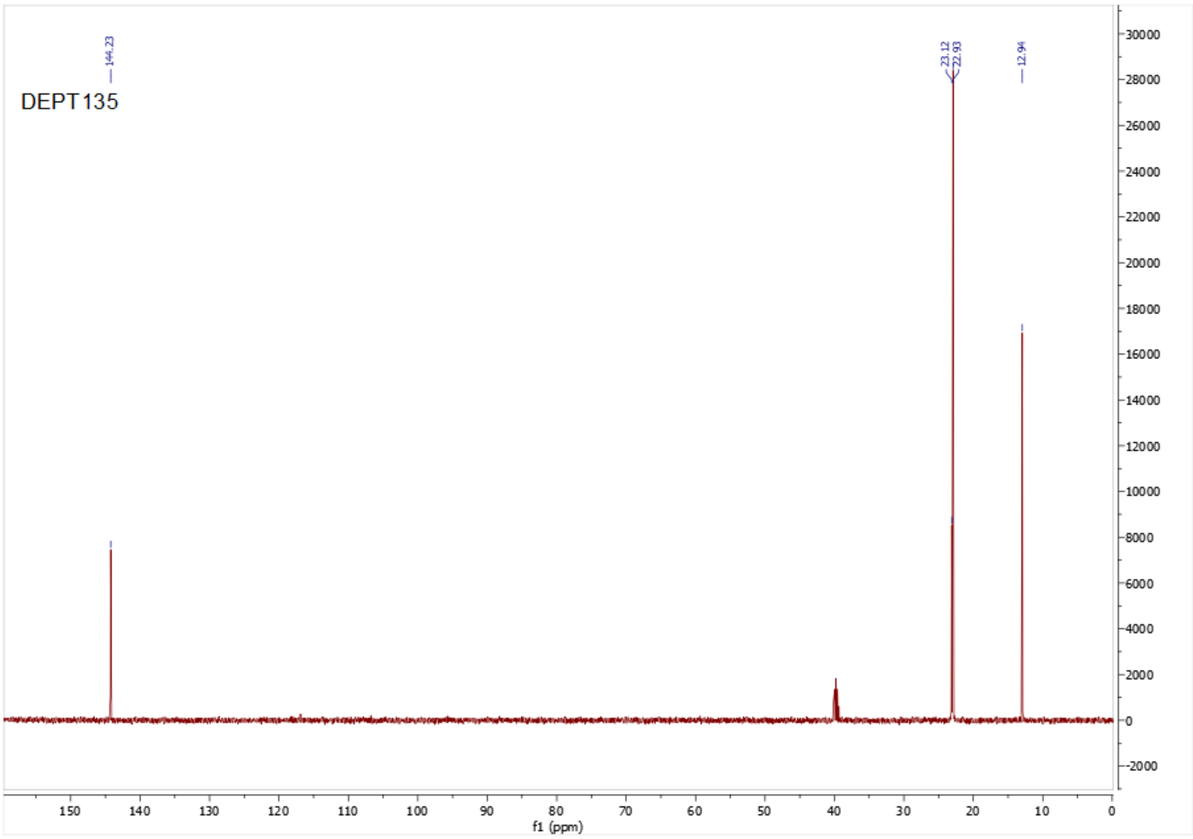

DEPT135
144.23
23.12
22.93
12.94
f1 (ppm)

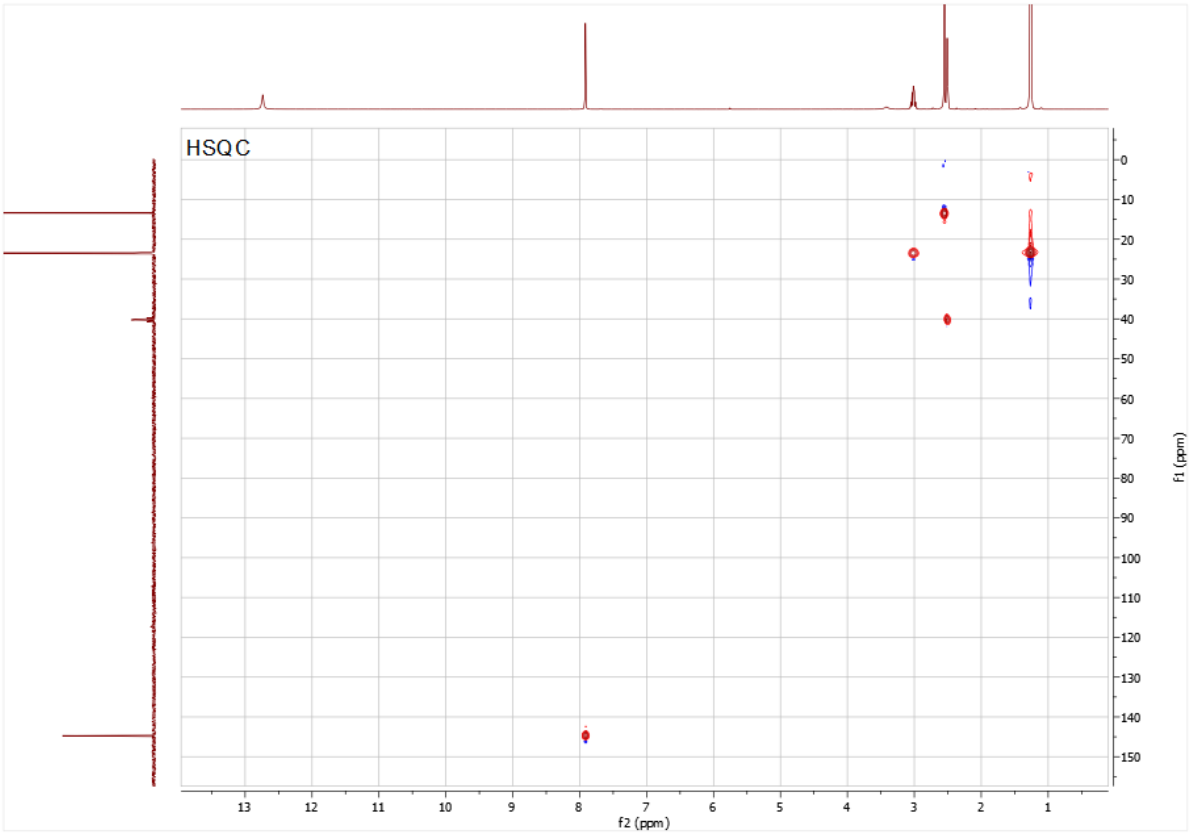

HSQC
f2 (ppm)
f1 (ppm)

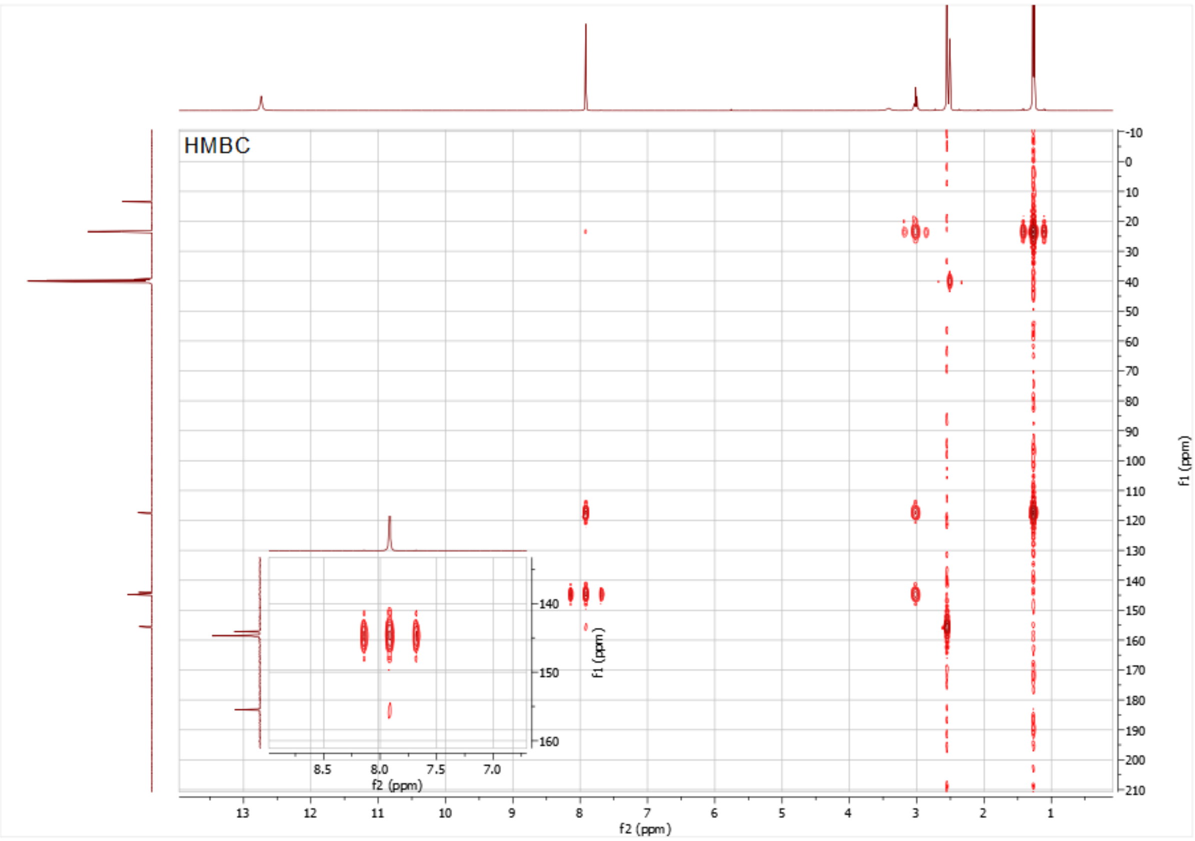
HMBC
f2 (ppm)
f1 (ppm)

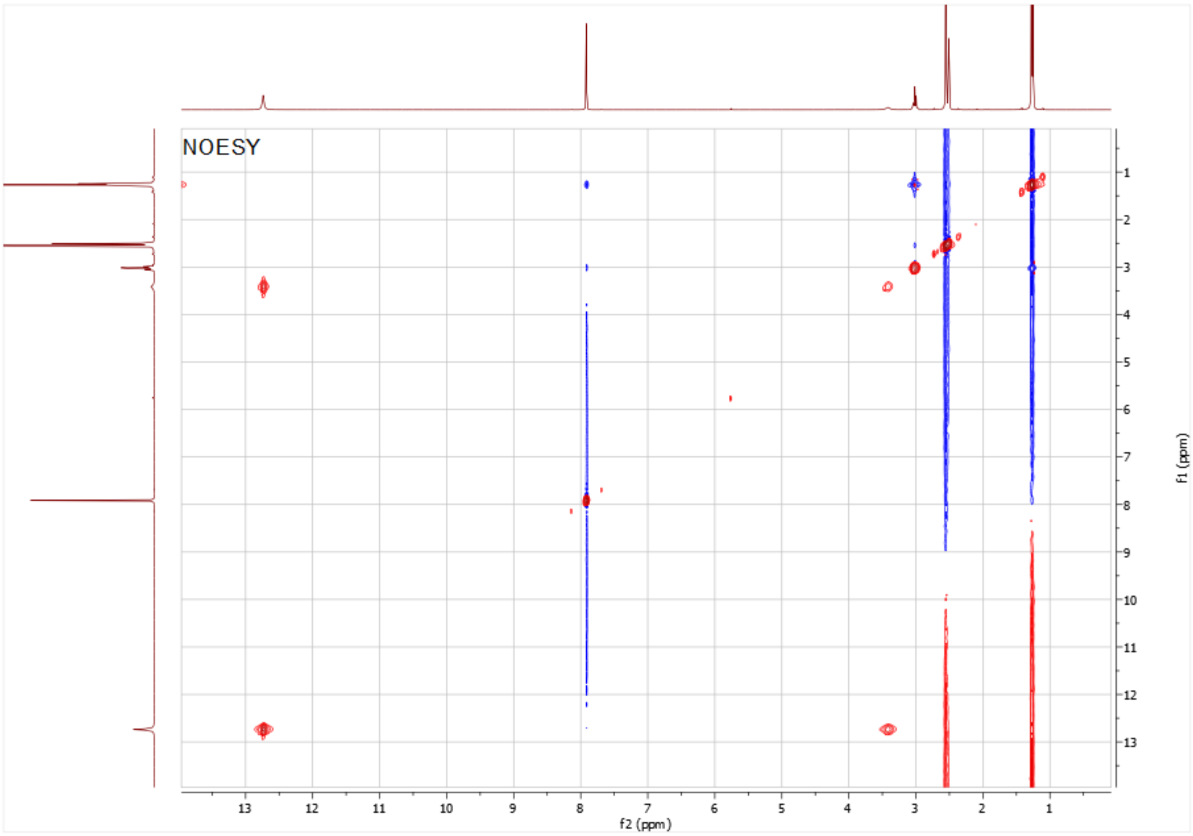
NOESY
f2 (ppm)
f1 (ppm)

8-Cyclopropyl-2-(methylsulfanyl)pyrazolo[1,5-a][1,3,5]triazin-4(3H)-one (**4h**)

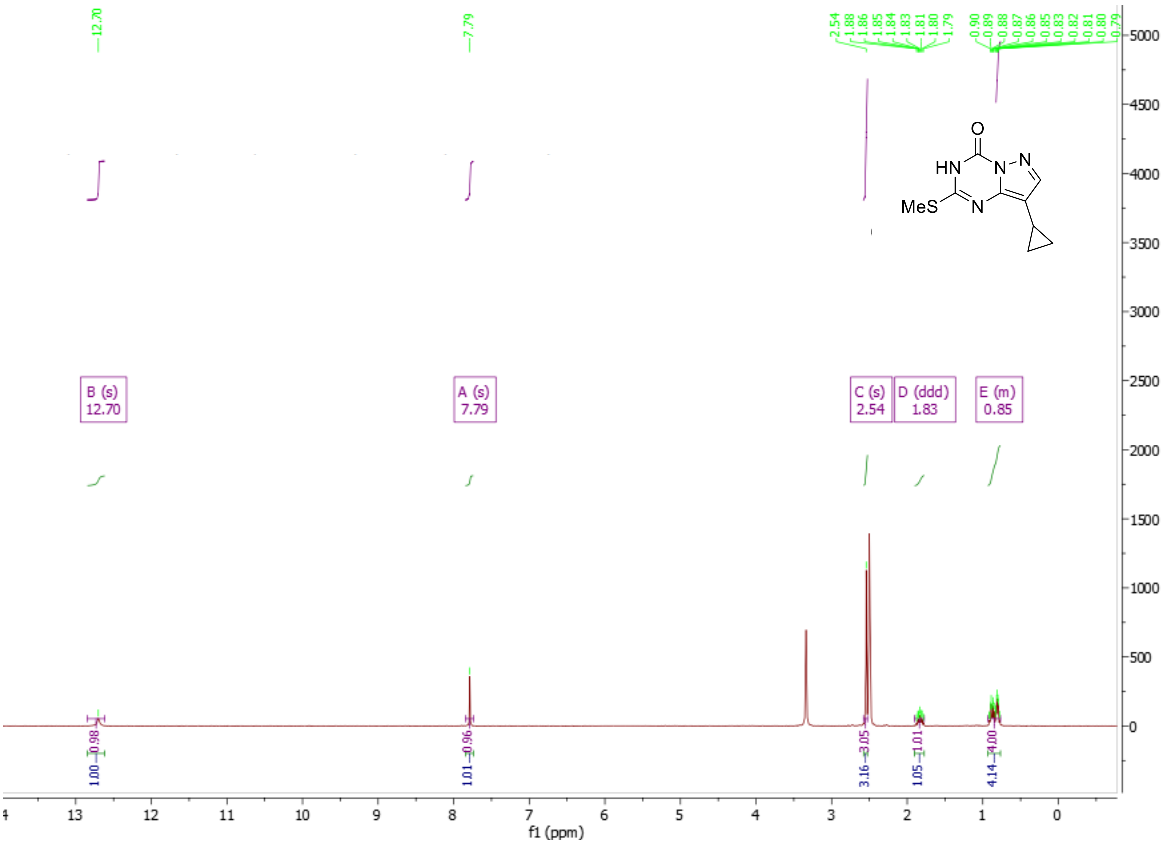

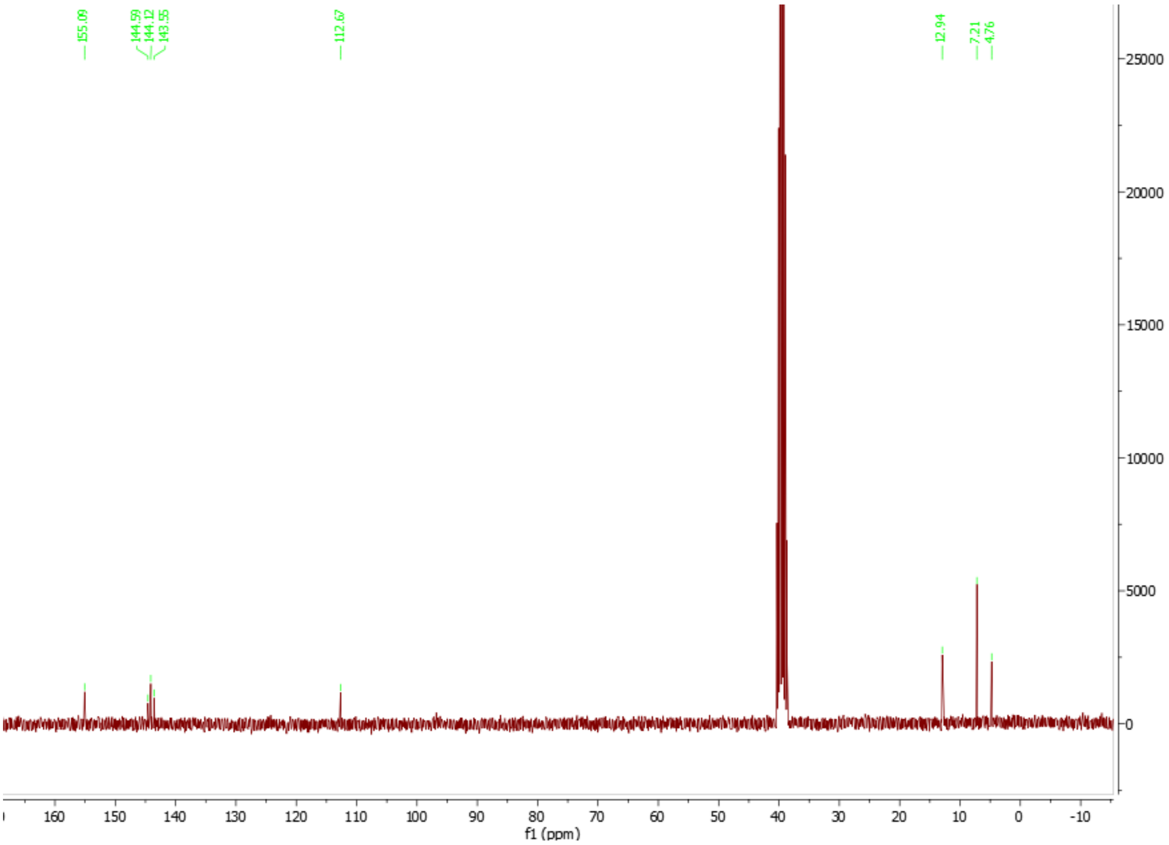

8-Cyclobutyl-2-(methylsulfanyl)pyrazolo[1,5-a][1,3,5]triazin-4(3H)-one (**4i**)

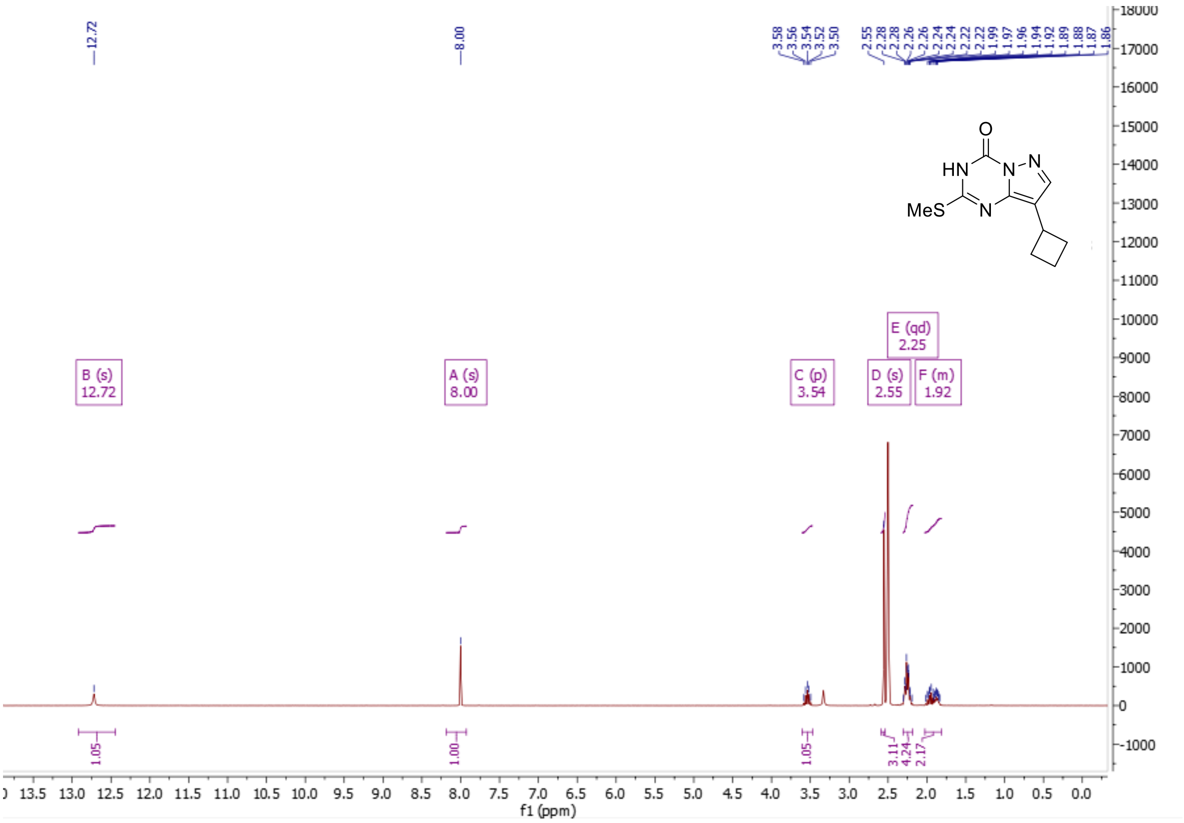

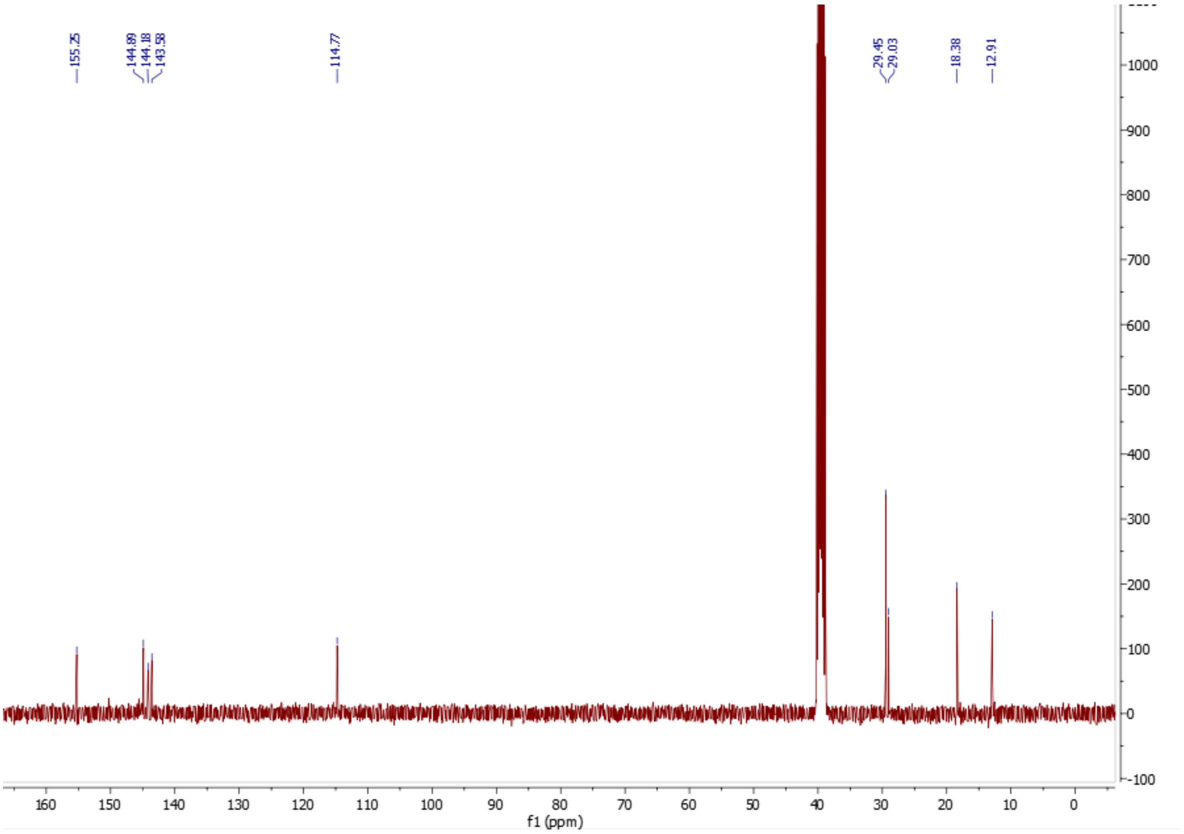

2-(Methylsulfanyl)-8-phenyl-pyrazolo[1,5-a][1,3,5]triazin-4(3H)-one (**4j**)

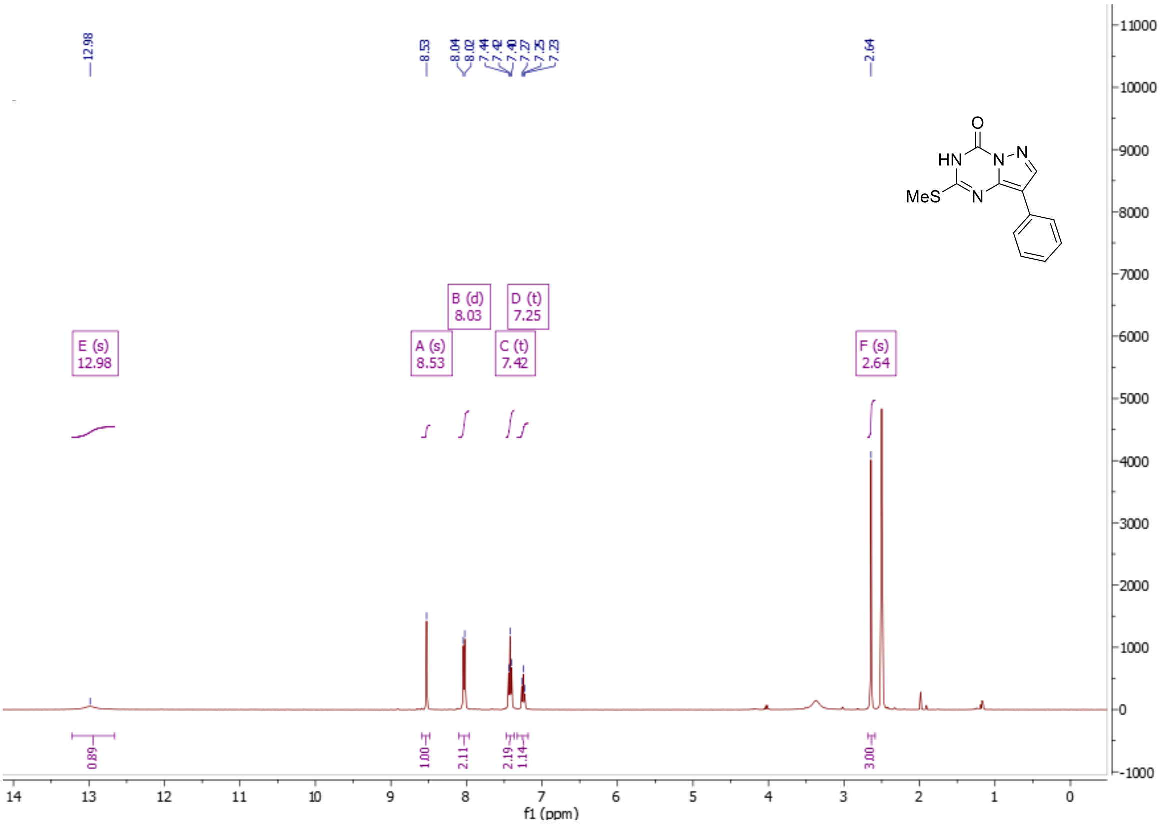

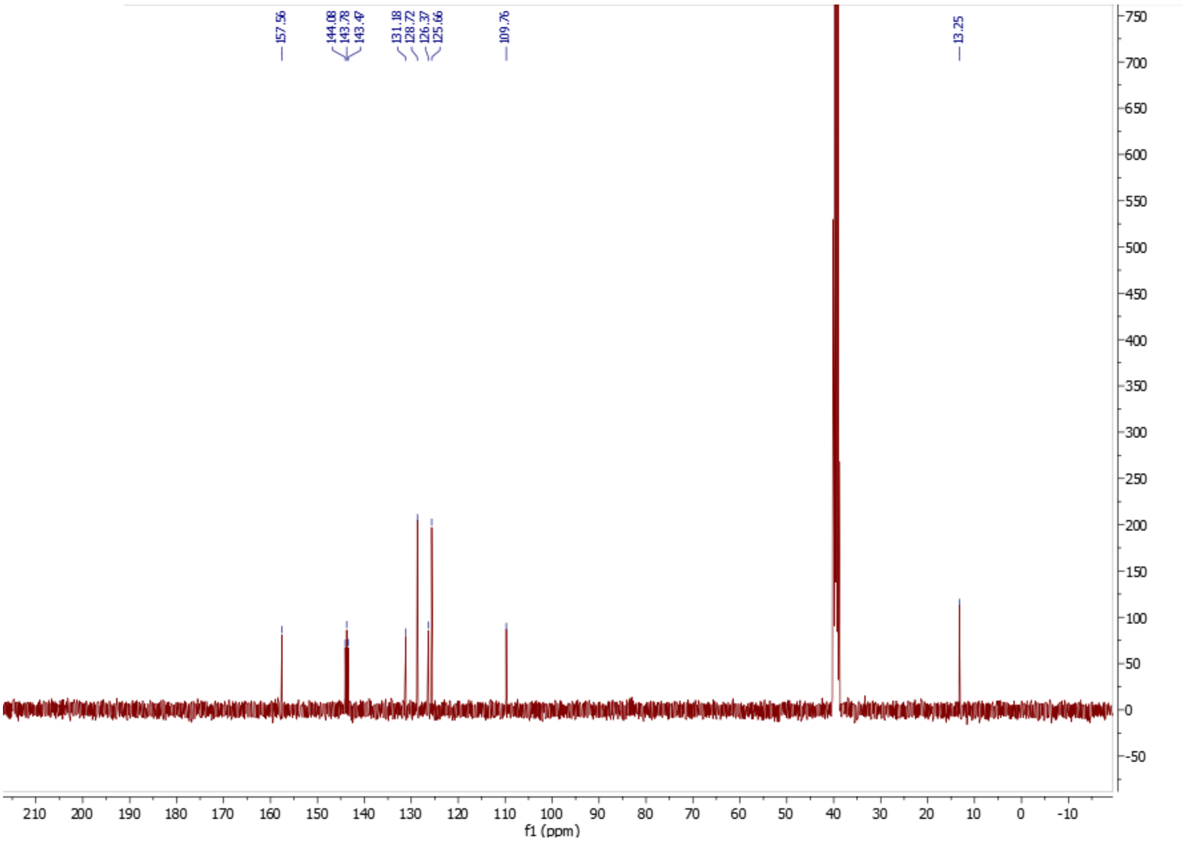

2-(Methylsulfanyl)-8-(p-tolyl)pyrazolo[1,5-a][1,3,5]triazin-4(3H)-one (**4k**):

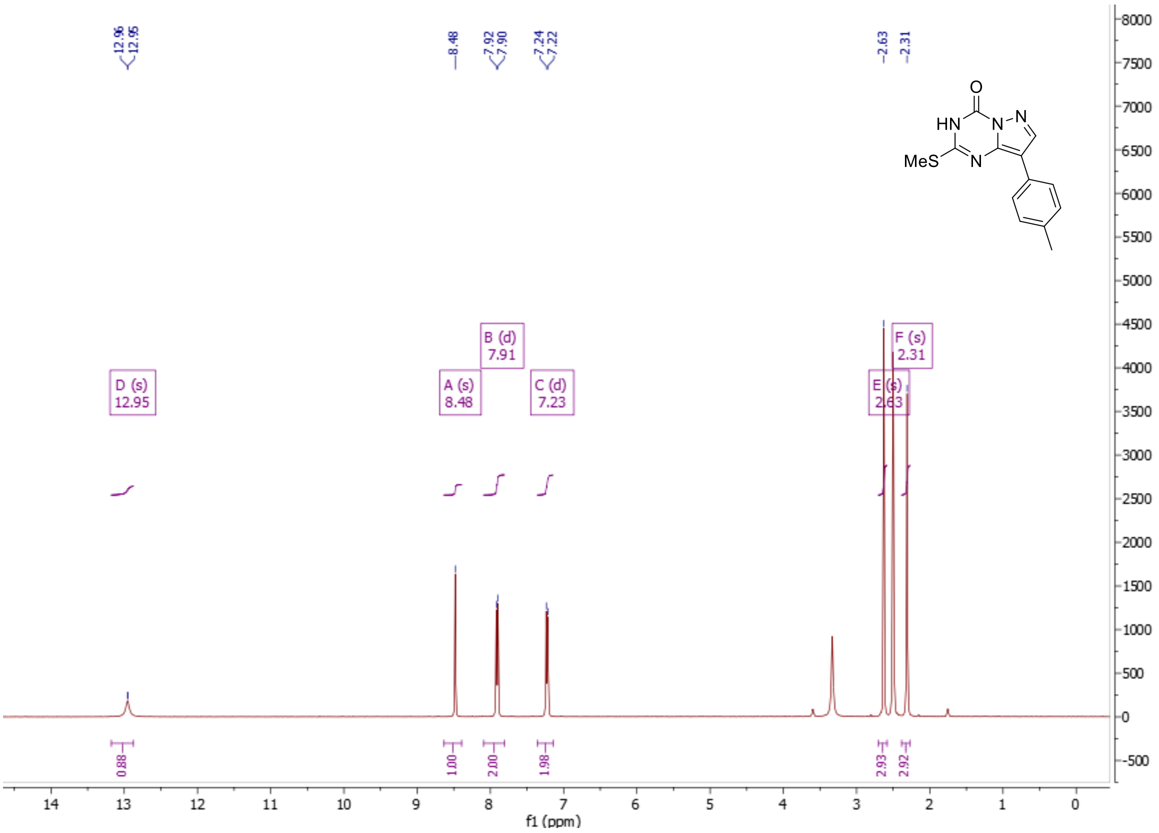

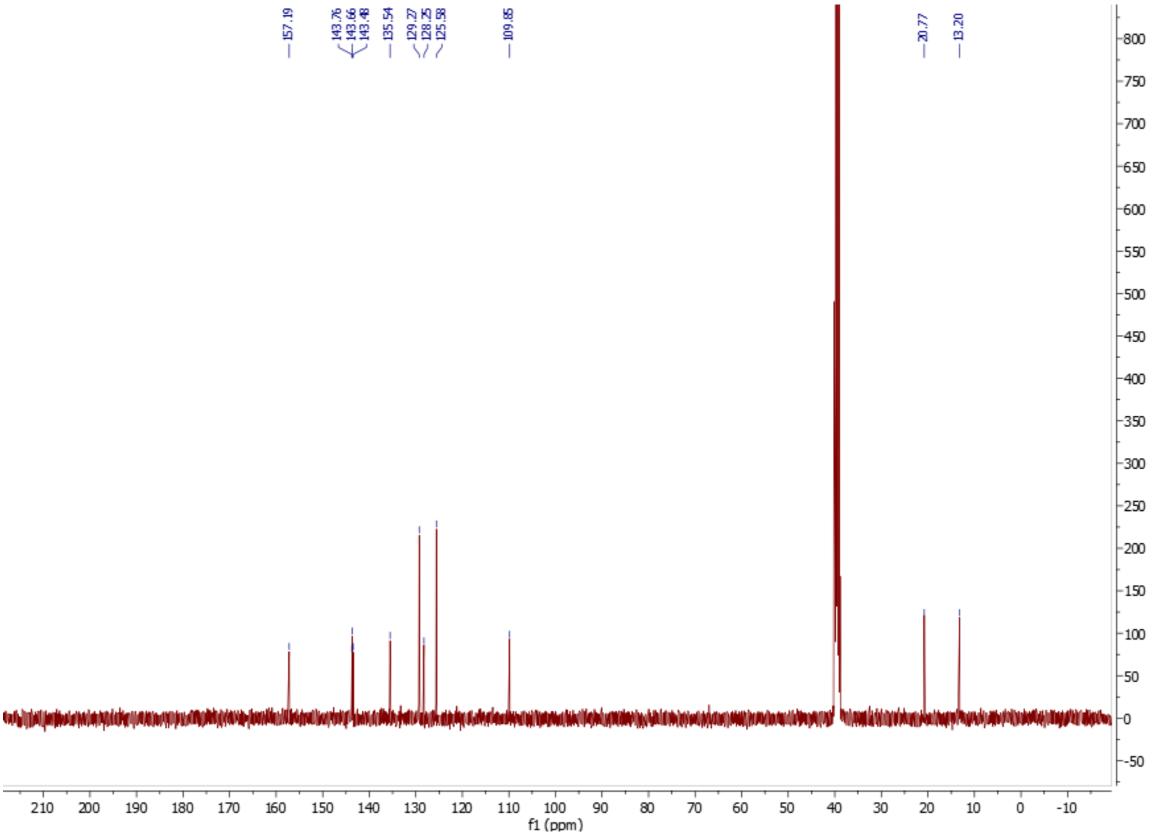

8-(4-Chlorophenyl)-2-(methylsulfanyl)pyrazolo[1,5-a][1,3,5]triazin-4(3H)-one (**4l**)

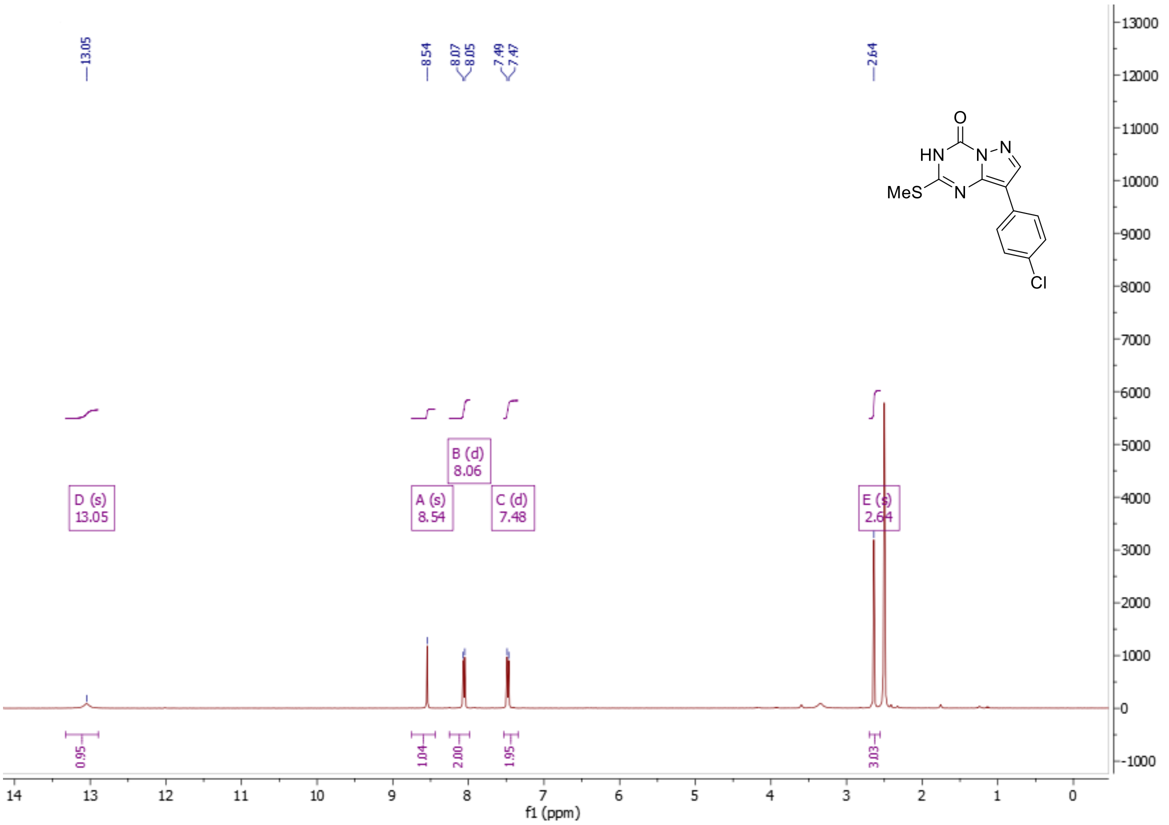

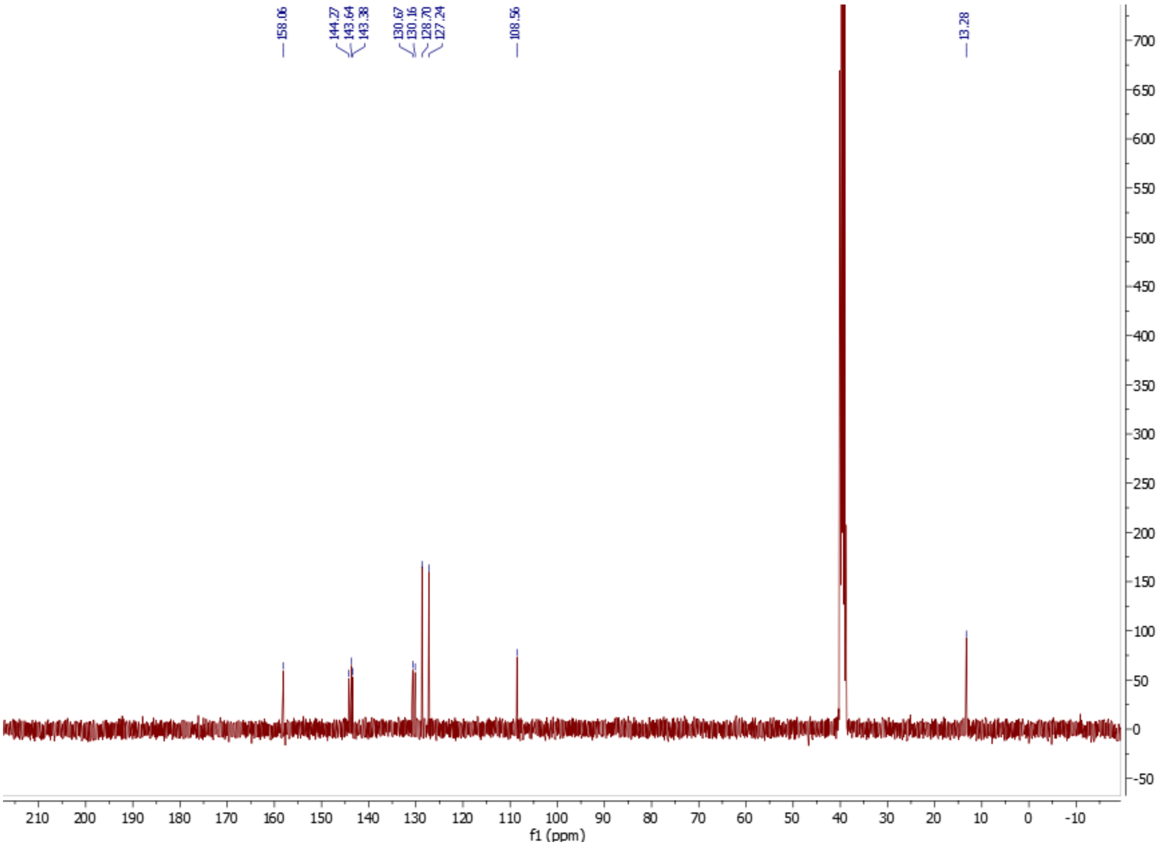

8-(4-Methoxyphenyl)-2-(methylsulfanyl)pyrazolo[1,5-a][1,3,5]triazin-4(3H)-one (**4m**)

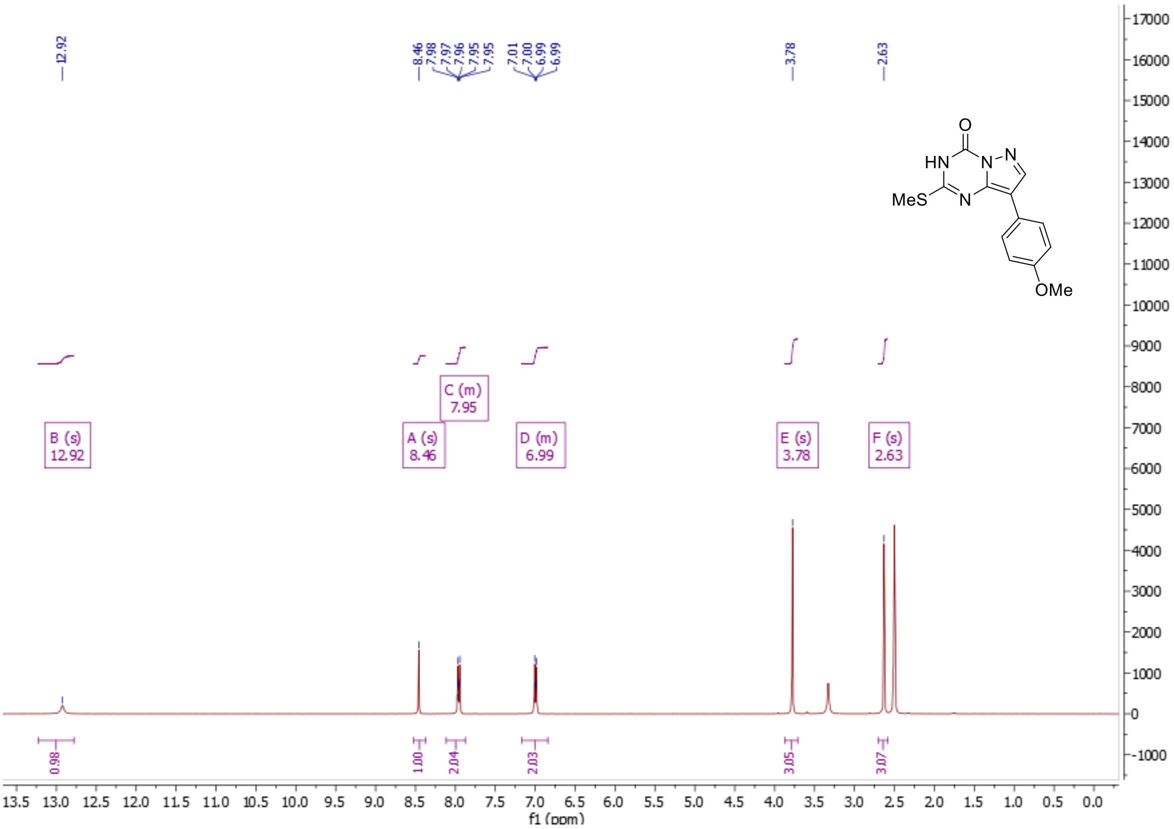

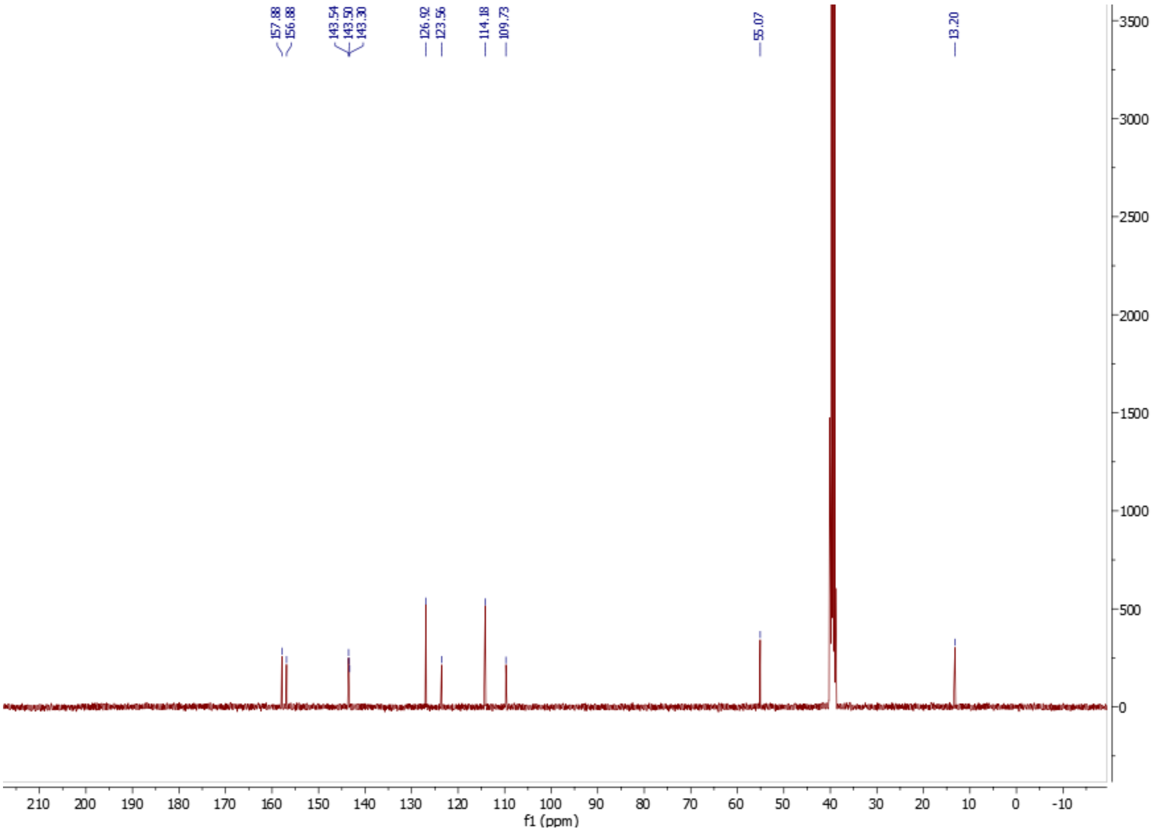

*2-(Methylsulfanyl)-8-(2-pyridyl)pyrazolo[1,5-a][1,3,5]triazin-4(3H)-one (**4n**)*

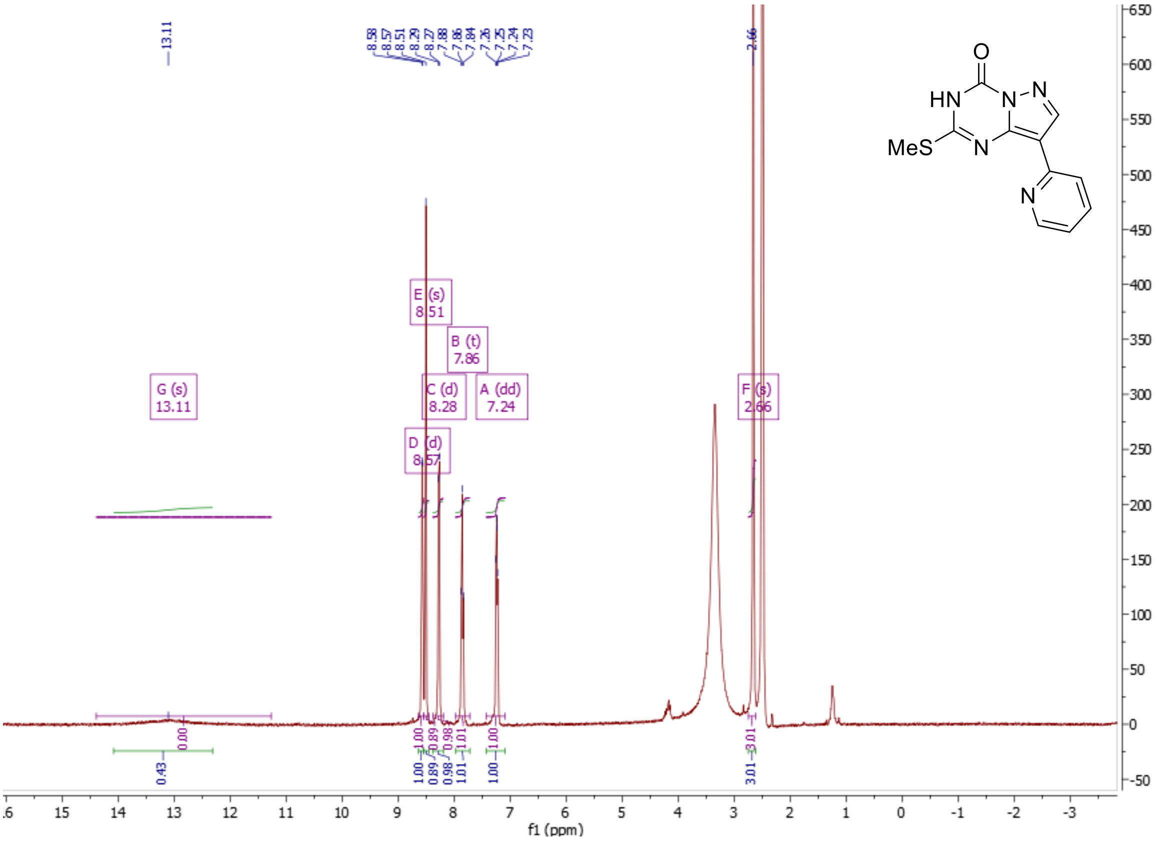

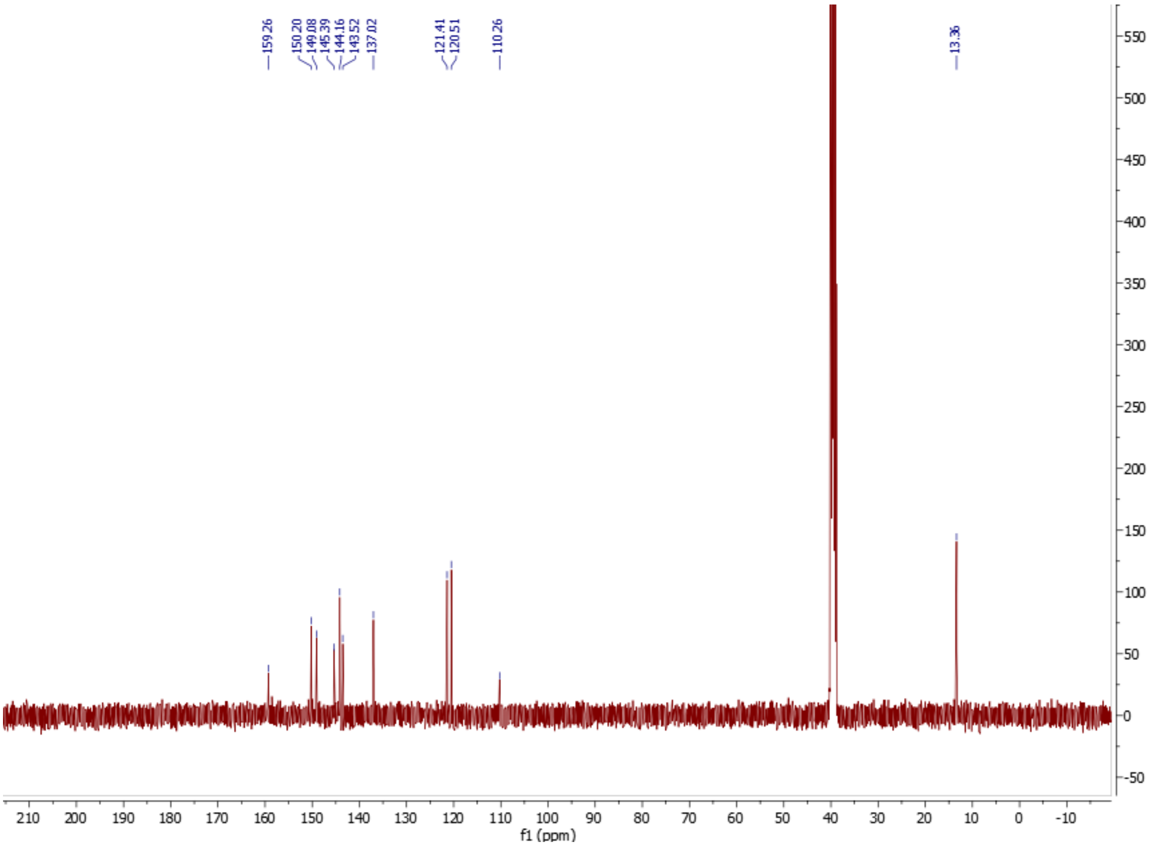

*2-thioxo-1H-pyrazolo[1,5-a][1,3,5]triazin-4-one (**3a**)*

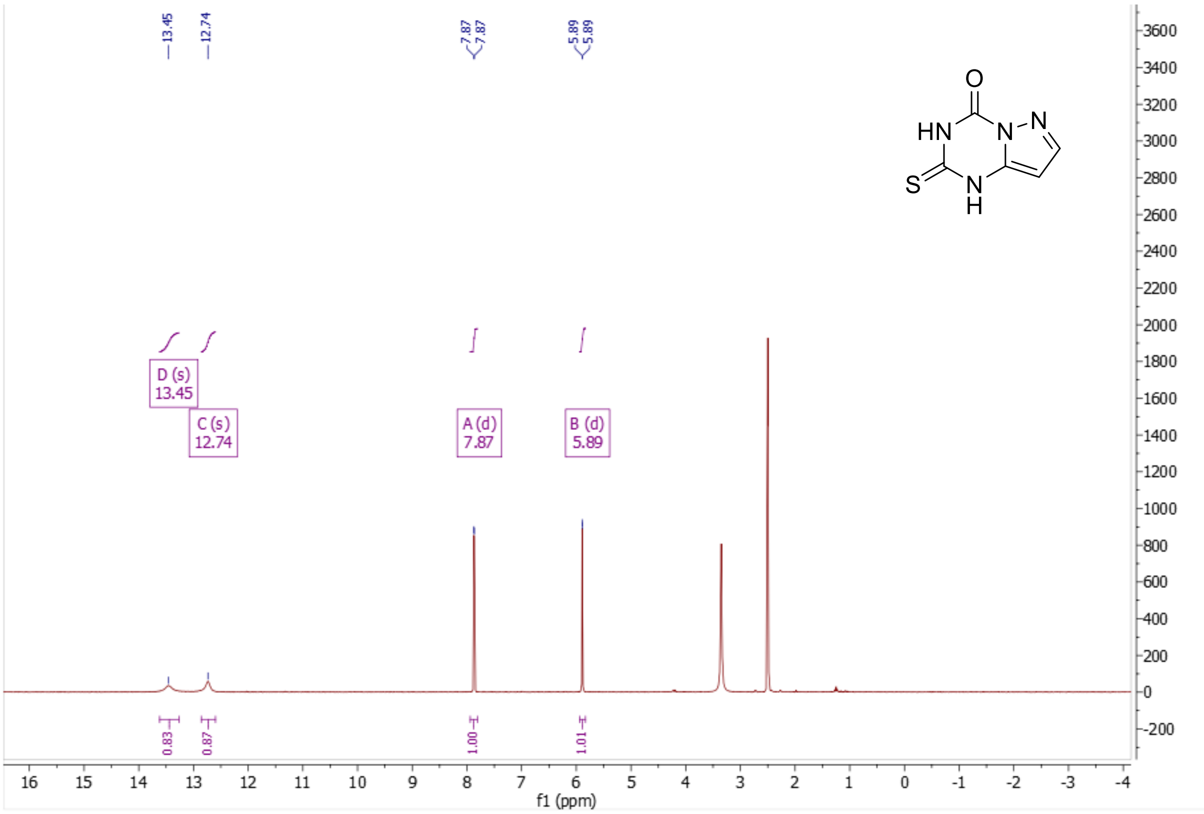

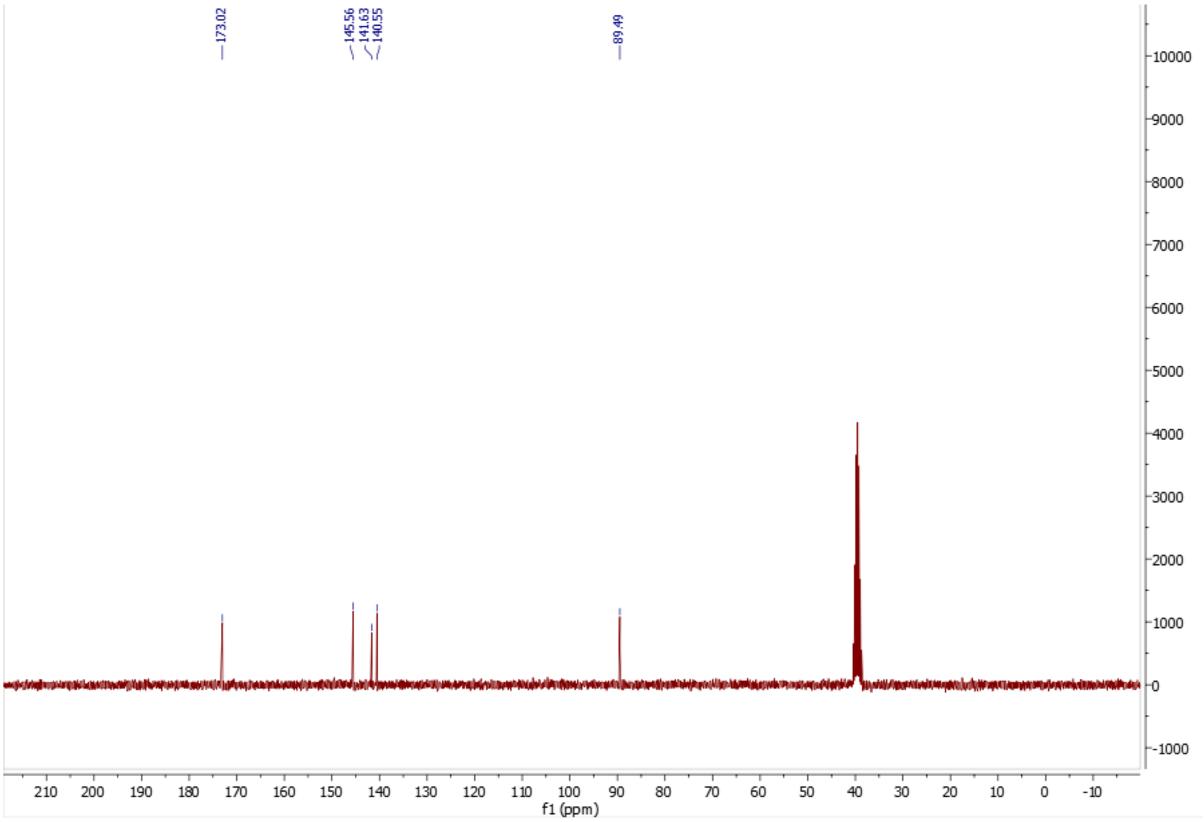

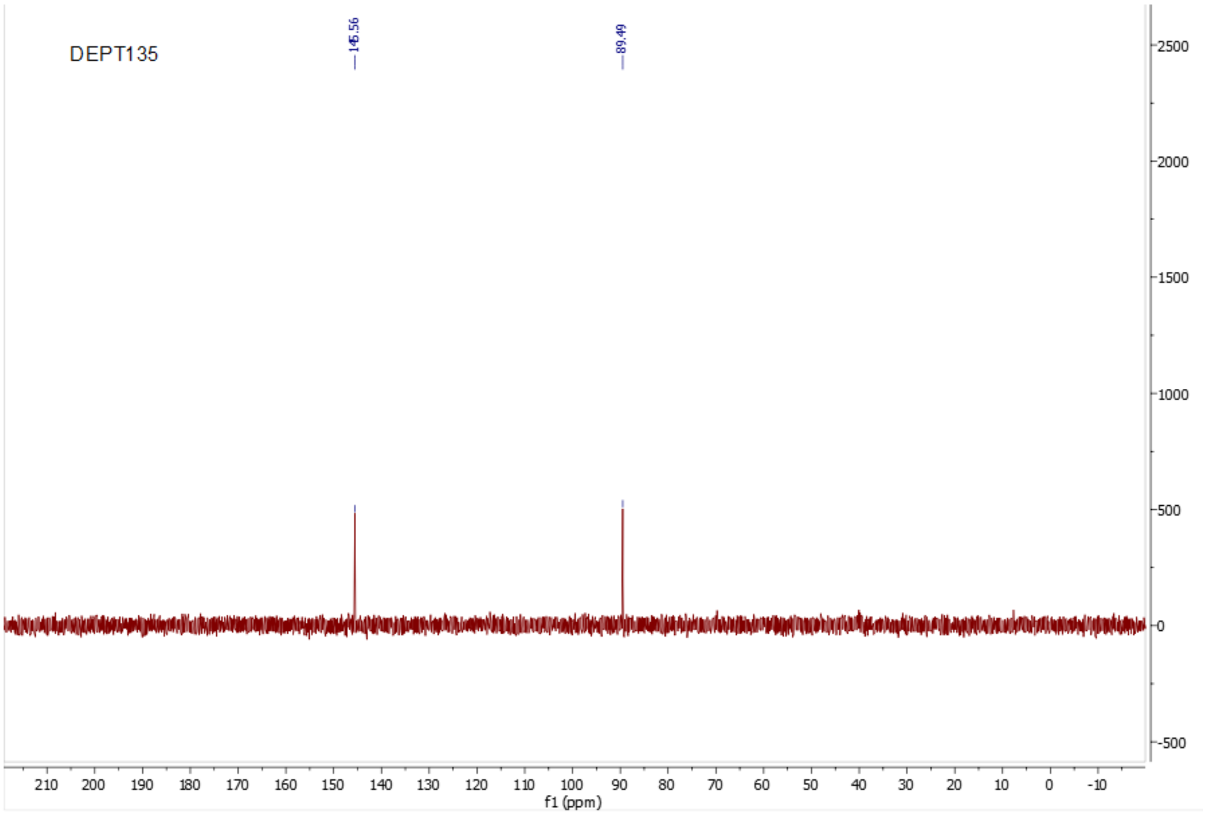
DEPT135
145.56
89.49
f1 (ppm)

*4-Chloro-2-(methylsulfanyl)pyrazolo[1,5-a][1,3,5]triazine (**5**)*

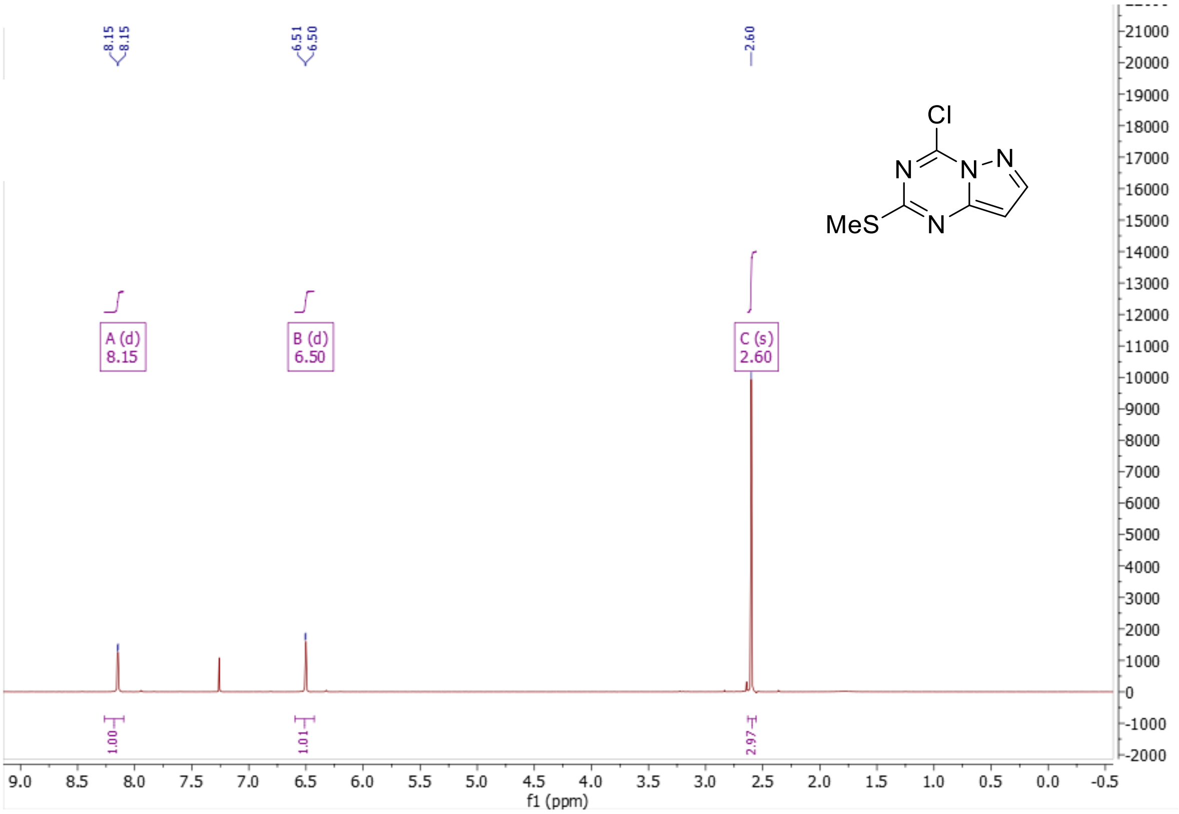

*8-Bromo-4-chloro-2-(methylsulfanyl)pyrazolo[1,5-a][1,3,5]triazine (**6**)*

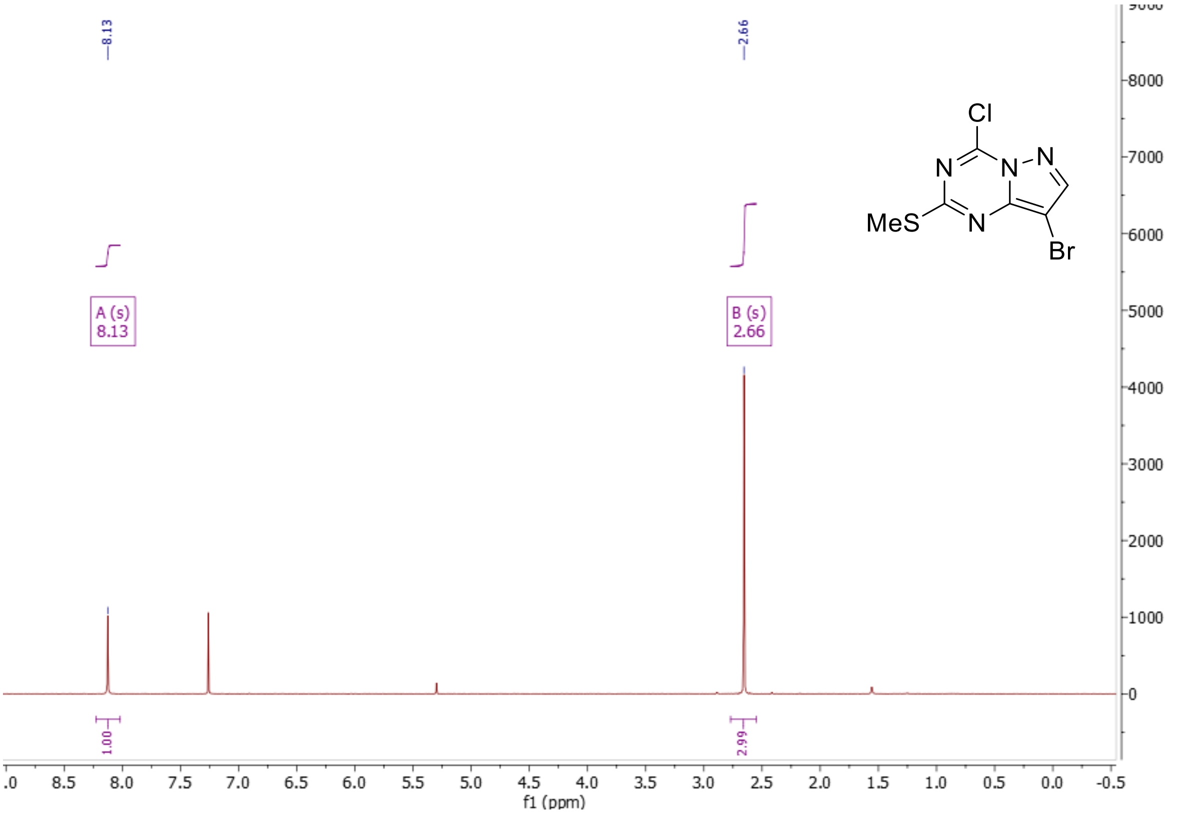

Supplement: Supplementary file 1 [file molecules-26-03540-s001.zip › molecules-1213301-supplementary.pdf]
